# Distinct metabolomic and proteomic signatures in Parkinson’s disease patients with REM sleep behavior disorder

**DOI:** 10.1038/s41392-026-02613-8

**Published:** 2026-03-30

**Authors:** Yaping Shao, Jing Wang, Yaping Liu, Yang Ni, Zijiao Liu, Yanli Li, Qiqi Jia, Qi Li, Xiaolin Wang, Tianbai Li, Meichen Liu, Shuining Zhang, Yanming Guo, Xisa Guo, Dali Wang, Yang Liu, Cong Liu, Huaibin Cai, Yuping Ning, Jihui Zhang, Guowang Xu, Weidong Le

**Affiliations:** 1 Key Laboratory of Liaoning Province for Research on the Pathogenic Mechanisms of Neurological Diseases, The First Affiliated Hospital, Dalian Medical University, Dalian, China; 2 Center for Sleep and Circadian Medicine, The Affiliated Brain Hospital, Guangzhou Medical University, Guangzhou, Guangdong China; 3 State Key Laboratory of Medical Proteomics, Dalian Institute of Chemical Physics, Chinese Academy of Sciences, Dalian, China; 4 Center for Clinical and Translational Medicine, Shanghai University of Medicine and Health Sciences, Shanghai, China; 5 Department of Clinical Laboratory, the First Affiliated Hospital of Dalian Medical University, Dalian, China; 6 Interdisciplinary Research Center on Biology and Chemistry, State Key Laboratory of Chemical Biology, Shanghai Institute of Organic Chemistry, Chinese Academy of Sciences, Shanghai, China; 7 Shanghai Academy of Natural Sciences (SANS), Fudan University, Shanghai, China; 8 Transgenic Section, Laboratory of Neurogenetics, National Institute on Aging, National Institutes of Health, Bethesda, MD USA; 9 Guangdong Engineering Technology Research Center for Translational Medicine of Mental Disorders, Guangdong, China; 10 Geriatric Neuroscience Center, The Affiliated Brain Hospital, Guangzhou Medical University, Guangzhou, Guangdong China

**Keywords:** Neurodevelopmental disorders, Predictive markers

## Abstract

Rapid eye movement sleep behavior disorder (RBD) is the most specific prodromal marker of Parkinson’s disease (PD), affecting 40–50% of PD patients. PD with RBD (RBD-PD) represents a clinically aggressive subtype characterized by more severe motor and nonmotor symptoms, prominent autonomic dysfunction, and accelerated disease progression; however, its underlying pathogenesis remains poorly understood. Here, we integrated multiplatform metabolomics and proteomics with precise clinical phenotyping to delineate molecular signatures in plasma across different PD subtypes. Our analyses demonstrated that PD patients exhibit significant metabolic reprogramming, characterized by a shift in energy metabolism from the tricarboxylic acid cycle toward glycolysis, a dysregulated urea cycle, and lipid remodeling, as well as extensive activation of inflammatory and immune responses involving the PI3K-Akt, IL-17, NF-kappaB, MAPK and TNF signaling pathways. Notably, the RBD-PD subgroup exhibited distinctive metabolic disturbances characterized by the accumulation of gut microbiota-derived toxic aromatic amino acid catabolites. Importantly, these alterations were also observed in idiopathic RBD (iRBD) patients, representing the prodromal stage of PD. By integrating metagenomic profiles, we further revealed that gut microbial dysbiosis in RBD-PD and iRBD drives a functional shift away from dietary fiber fermentation and toward enhanced degradation of protein, aromatic amino acids, glycine, and intestinal mucin glycans. This metabolic reprogramming is associated with exacerbated oxidative stress, neuroinflammation, and accelerated pathological progression. These findings provide multiomic evidence that clarifies the molecular heterogeneity in PD and highlights gut microbiota-driven dysfunction as a key contributor to both the iRBD and RBD-PD subtypes.

## Introduction

Parkinson’s disease (PD) is a progressive neurodegenerative disorder characterized by selective nigrostriatal dopaminergic loss and the formation of intraneuronal Lewy bodies resulting from aberrant α-synuclein aggregation.^1,2^ Although the clinical diagnosis of PD relies on the appearance of motor manifestations, extensive neuropathological dissemination initiates decades earlier, posing a significant challenge for early diagnosis.^3^ Rapid eye movement sleep behavior disorder (RBD) represents the most specific prodromal marker of PD, conferring over 90% risk of phenoconversion to α-synucleinopathies, predominantly PD, over a 14-year follow-up period.^4,5^ In established PD, RBD also represents a prevalent nonmotor manifestation, affecting approximately 40 ~ 50% of patients.^6^ Extensive studies have demonstrated that PD patients with RBD (RBD-PD) exhibit more severe motor and nonmotor symptoms, including pronounced autonomic dysfunction, earlier cognitive deterioration, and accelerated motor progression.^7,8^ RBD is typically persistent in PD patients and may worsen over time.^9^ For example, one longitudinal study reported that probable RBD symptoms disappeared in only 7.5% of PD patients during a four-year follow-up.^10^ These findings highlight the substantial heterogeneity inherent in PD pathophysiology and support RBD-PD as an aggressive clinical subtype.^8^ However, its underlying pathogenic mechanisms remain incompletely understood.

One possible explanation for the clinical differences between RBD-PD and PD without RBD (non-RBD-PD) is the “body-first” and “brain-first” hypotheses of PD pathogenesis.^11,12^ This model proposes that the RBD-PD subtype follows a “body-first” trajectory, in which α-synuclein pathology originates in the peripheral or enteric nervous system (ENS) and subsequently ascends via the vagus nerve to the brainstem, particularly the locus coeruleus/subcoeruleus complex, which is considered one of the core pathological nuclei involved in RBD.^12^ The pathology then continues to propagate rostrally to more central structures, such as the substantia nigra, leading to the emergence of motor symptoms.^12^ Conversely, non-RBD-PD may reflect a “brain-first” subtype, in which pathological α-synuclein originates within the central nervous system and then spreads peripherally.^12^ This hypothesis reframes the clinical distinction between RBD-PD and non-RBD-PD as potentially rooted in divergent pathogenic origins and provides a falsifiable biological framework for mechanistic investigation.^13^

Several lines of evidence support this hypothesis. PD patients with RBD exhibit increased phosphorylated α-synuclein pathology within the ENS.^14,15^ Similarly, patients manifesting prodromal RBD demonstrate a higher prevalence of peripheral neurodegeneration, including severe constipation and enteric α-synuclein deposition.^1,16^ PD-like gut dysbiosis has been identified at both the prodromal stage and in RBD-PD patients.^1,17^ It has been reported that the reduction of butyrate-producing bacteria and the enrichment of proinflammatory *Collinsella* are observed in both idiopathic RBD (iRBD) patients and their first-degree relatives.^1^ Compared with non-RBD-PD patients, those with RBD-PD exhibit increased abundances of opportunistic pathogens (e.g., *Aerococcus*) and decreased abundances of beneficial taxa (e.g., *Butyricicoccus* and *Faecalibacterium*).^13^ These findings suggest that gut microbiota dysbiosis and associated inflammatory cascades might contribute to the divergent pathomechanisms underlying distinct PD subtypes; however, the specific molecular pathways mediating this gut-brain crosstalk remain poorly understood. The gut microbiota modulates host homeostasis and physiological processes primarily through microbe-derived metabolites, which regulate systemic inflammation and neuronal function. Our previous study revealed significant metabolic dysregulation in PD patients, including elevated levels of neurotoxic bile acids (cholic acid and deoxycholic acid) and decreased levels of neuroprotective ursodeoxycholic acid (UDCA) and tauroursodeoxycholic acid (TUDCA).^18^ Notably, fecal microbiota transplantation (FMT) from established PD patients exacerbated inflammation and neurodegeneration in A53T mice.^19^ Compared to FMT from healthy controls, FMT from PD patients significantly reduced fecal UDCA levels and increased the abundances of proinflammatory bacteria such as *Alloprevotella*, *Flavonifractor*, *Erysipelatoclostridium*, and *Subdoligranulum* in A53T mice.^19^ Moreover, a recent study revealed that the gut microbiota-derived metabolites, such as secondary bile acids and *p*-cresol sulfate, were enriched in PD patients with RBD.^20^ These findings highlight the critical role of gut microbiota‒metabolite interactions in PD pathophysiology. Nevertheless, direct comparative investigations between RBD-PD patients and non-RBD-PD patients are critically limited.

**Fig. 1:**
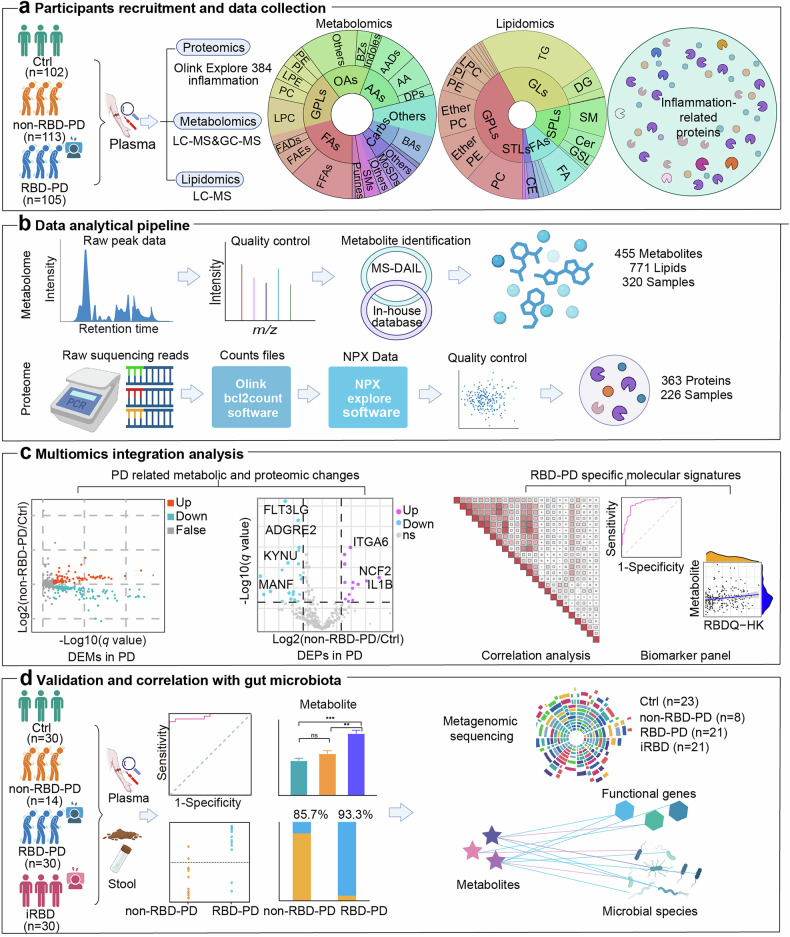
Flowchart of subject recruitment and integrated analysis of plasma metabolomics and proteomics in PD patients. **a** Multiplatform metabolomics and proteomics were performed to profile plasma molecular changes in patients with RBD-PD, non-RBD-PD patients and healthy controls. **b** A total of 455 polar metabolites, 771 lipid species, and 363 inflammation- and immune-related proteins were annotated. **c** By integrating multiomics data, PD-related metabolic and proteomic changes as well as RBD-PD-specific molecular signatures were identified. **d** Following the validation of the RBD-PD-specific metabolic changes in an independent clinical cohort, we integrated metagenomic data to identify the gut microbes and functional genes associated with these specific, microbially derived metabolites. Ctrl healthy control, non-RBD-PD PD patients without RBD; RBD-PD PD patients with RBD, iRBD idiopathic RBD, FAs fatty acyls, GLs glycerolipids, GPLs glycerophospholipids, SPLs sphingolipids, STLs sterol lipids, OAs organic acids, AAs amino acids, Carbs carbohydrates, FFAs fatty acids, FAEs fatty acid esters, FADs fatty amides, LPC lysophosphatidylcholine, PC phosphatidylcholine, PI phosphatidylinositol, PE phosphatidylethanolamine, LPE lysophosphatidylethanolamine, BZs Benzene and substituted derivatives, AADs amino acids derivatives, DPs dipeptides, BAs bile acids, SMs sphingomyelins, CE cholesterol ester, GSL glycosphingolipid, DG diacylglycerol, TG triacylglycerol

In this study, we initially recruited PD patients with and without RBD, as well as matched healthy controls. Plasma samples from all participants were subjected to metabolomic and lipidomic analyses via liquid chromatography‒mass spectrometry (LC‒MS) and gas chromatography‒mass spectrometry (GC‒MS) platforms. Additionally, targeted profiling of inflammatory and immune-associated proteins was performed on the majority of these plasma samples. By integrating metabolomic and proteomic data, we identified both shared and subtype-specific molecular signatures across the two PD subtypes. We further elucidated the correlations between key metabolites and proteins, as well as their associations with clinical characteristics. Notably, key metabolic alterations observed in RBD-PD patients were subsequently validated in an independent cohort comprising iRBD patients, PD patients with and without RBD, and healthy individuals. The relationship between these metabolites and gut microbiota dysbiosis was also investigated (Fig. 1). To our knowledge, this study represents the largest comparative analysis of these PD subtypes to date. By revealing subtype-specific molecular and biological pathways, we aim to identify candidate biomarkers and elucidate the distinct molecular mechanisms that may contribute to the heterogeneous clinical trajectories of PD.

## Results

### Participant cohorts and study design

This study enrolled 424 participants from two independent cohorts comprising 135 RBD-PD patients, 127 non-RBD-PD patients, 30 iRBD patients and 132 healthy controls (Ctrl). In the discovery phase (Cohort 1), RBD status was evaluated via validated questionnaires (RBDQ-HK, cutoff of 18 for the diagnosis of RBD, detailed in the Methods section, Supplementary Fig. 1). The three groups, including the non-RBD-PD, RBD-PD and Ctrl groups, demonstrated comparable age (*p* = 0.0632, one-way ANOVA) and sex distributions (*p* = 0.9664, χ² test). Patients with RBD-PD exhibited significantly greater motor impairment (Hoehn-Yahr stage, mean: 2.7 vs. 2.2; *p* = 0.0002), poorer sleep quality (PSQI mean: 11.4 vs. 9.1; *p* = 0.0005), and more severe daytime sleepiness (ESS mean: 10.4 vs. 7.7; *p* = 0.0009) than non-RBD-PD patients.

In the validation stage, an independent cohort (Cohort 2) comprising four age- and sex-matched groups (30 RBD-PD patients, 14 non-RBD-PD patients, 30 iRBD patients and 30 Ctrl subjects, all of whom underwent gold-standard video polysomnography (vPSG) assessment for the confirmation of RBD status) was included. As expected, we observed more severe RBD symptoms in both the RBD-PD and iRBD groups than in the non-RBD-PD group, despite seemingly comparable daytime sleepiness between non-RBD-PD patients and RBD-PD patients **(**Table 1**)**.

**Table 1:** Demographic characteristics of the subjects

	Cohort 1				Cohort 2				
	Ctrl (*n* = 102)	non-RBD-PD (*n* = 113)	RBD-PD (*n* = 105)	*p*-value	Ctrl (*n* = 30)	non-RBD-PD (*n* = 14)	RBD-PD (*n* = 30)	iRBD (*n* = 30)	*p*-value
Age, years, mean (SD)	66.4 (7.0)	65.9 (8.1)	68.2 (7.0)	0.0632	63.1 (7.0)	62.8 (11.2)	64.8 (8.3)	64.8 (6.0)	0.7137
Female/Male	54/48	58/55	54/51	0.9664	9/21	4/10	8/22	8/22	0.9901
Disease duration, years, mean (SD)	—	5.4 (5.0)	7.5 (5.0)	0.0002	—	4.6 (2.9)	3.4 (3.4)	—	0.1402
H-Y staging, mean (SD)	—	2.2 (0.8)	2.7 (0.7)	0.0002	—	1.9 (1.4)	1.8 (1.1)	—	0.9647
RBDQ-HK, mean (SD)	—	7.4 (5.0)	34.5 (10.9)	<0.0001	4.5 (5.1)	13.9 (18.1)	33.9 (23.1)	47.1 (13.7)	<0.0001
PSQI, mean (SD)	—	9.1 (4.4)	11.4 (4.5)	0.0005	—	—	—	—	—
ESS, mean (SD)	—	7.7 (4.8)	10.4 (5.8)	0.0009	15.6 (5.2)	18.1 (4.6)	17.2 (4.9)	13.5 (3.6)	0.0045
History of diabetes, Y/N	22/80	15/98	14/91	0.1700	—	—	—	—	—
Hyperlipidemia, Y/N	17/85	19/94	16/89	0.9425	11/19	2/12	10/20	5/25	0.1859
Anxiety or depression, Y/N	—	21/92	21/84	0.7911	3/27	5/9	9/21	2/28	0.0213
Anti-PD drugs, Y/N	—	87/26	91/14	0.0652	—	12/2	25/5	—	0.8406

Differences in age distribution among the groups were calculated via one-way ANOVA. Differences in sex distribution, history of diabetes, hyperlipidemia, anxiety or depression and anti-PD drugs were calculated via chi-square tests. For other continuous variables, differences between two groups were assessed via the Mann‒Whitney test, whereas differences among multiple groups were assessed via one-way ANOVA. *Y/N* yes/no, *Ctrl* healthy control, *non-RBD-PD* PD patients without RBD, *RBD-PD* PD patients with RBD, *iRBD* idiopathic RBD, *H-Y* Hoehn-Yahr, *PSQI* Pittsburgh Sleep Quality Index, *RBDQ-HK* REM sleep behavior disorder questionnaire–Hong Kong, *ESS* Epworth sleepiness scale

### Plasma metabolic profiling and quality control

In Cohort 1, plasma samples were subjected to comprehensive metabolomics analysis via both GC‒MS and LC‒MS platforms. This integrated approach identified 455 unique metabolite features (Supplementary Tables 1, 2). We additionally performed lipidomic analysis on 314 (Ctrl, *n* = 102; non-RBD-PD, *n* = 110; RBD-PD, *n* = 102) of the 320 plasma samples, identifying a total of 771 annotated lipid species (Supplementary Table 3**)**. The identified lipids spanned five major categories: fatty acyls, glycerolipids (GLs), glycerophospholipids (GPLs), sphingolipids (SPLs), and sterol lipids (STLs). These categories encompassed 27 distinct subclasses as defined by the LIPID MAPS classification system.

To ensure the stability of the entire analytical process and monitor instrument reproducibility, quality control (QC) samples were prepared and inserted throughout the analytical sequence (detailed in the Supplementary Materials). After calibration using multiple internal standards (ISs, detailed in the Supplementary Materials), the QC samples exhibited tight clustering on the principal component analysis (PCA) score plot. Moreover, more than 95% of the metabolites and 87% of the lipids achieved a relative standard deviation (RSD) of less than 30% in the QC samples. These results demonstrated minimal variability and robustness of the data generated from all three platforms (Supplementary Fig. 2).

### Metabolic signatures of PD patients

**Fig. 2:**
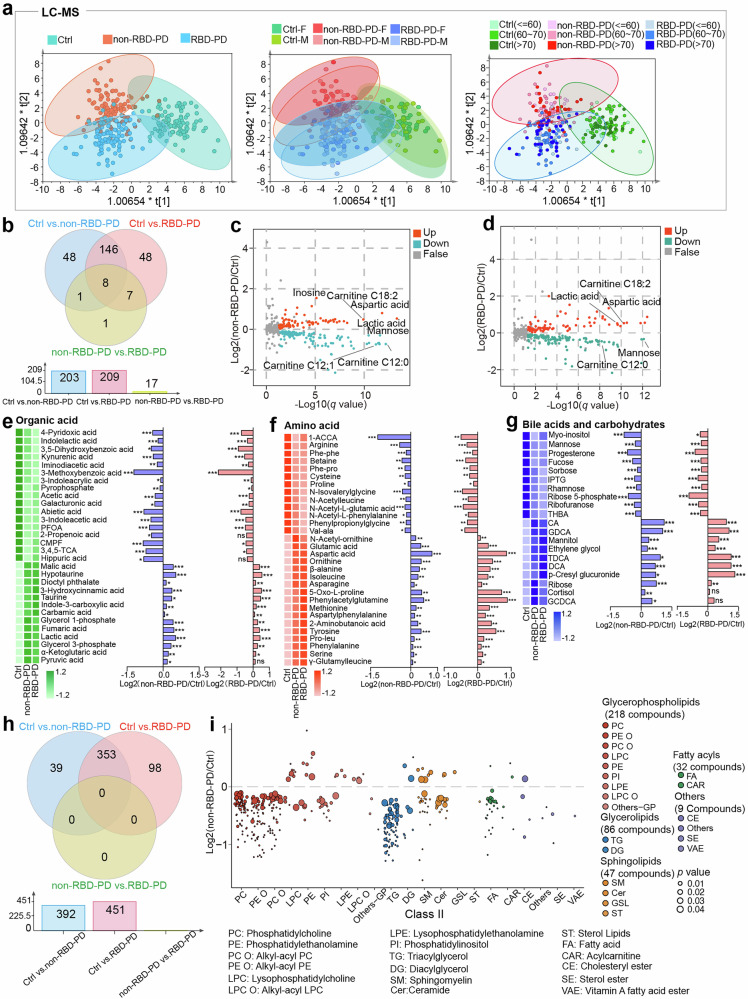
Identification of differentially expressed metabolites and lipids in PD. **a** OPLS‒DA model of two PD subgroups and control individuals on the basis of metabolic profiles via LC‒MS (R2Y = 0.654, F factor = 11.36, *p*-value = 0). **b** Venn diagram illustrating the number of differentially abundant metabolites identified in the between-group comparisons. Statistical significance between two groups was assessed via the Mann‒Whitney test, and the resulting *p* values were adjusted for multiple comparisons via the Benjamini‒Hochberg procedure. **c** Volcano plot visualizing significantly changed differentially abundant metabolites in non-RBD-PD patients compared with Ctrl individuals. **d** Volcano plot visualizing significantly changed differentially abundant metabolites in the RBD-PD group compared with the Ctrl group. **e**–**g** Heatmaps of the relative concentrations of non-RBD-PD-related differentially abundant metabolites (e.g., organic acids, amino acids, bile acids and carbohydrates) and fold changes between the two PD subgroups and healthy controls. *0.01 < *q* < 0.05, **0.001 < *q* < 0.01, ***q < 0.001. The *q* value represents the FDR-adjusted *p* value for multiple hypothesis testing. To better visualize the relative concentrations of the mean values of each metabolite in the different groups, Z score scaling was performed. The color scale in the heatmap represents the Z score. **h** Venn diagram indicating the number of differential lipids in between-group comparisons (based on Mann‒Whitney tests and multiple comparisons correction). **i** Bubble plot of fold changes for differential lipids of non-RBD-PD patients categorized by chemical class. PFOA perfluorooctanoic acid, CMPF 3-carboxy-4-methyl-5-propyl-2-furanpropionic acid, 3,4,5-TCA 3,4,5-trimethoxycinnamic acid, 1-ACCA 1-aminocyclohexane carboxylic acid, Phe-phe phenylalanyl-phenylalanine, Phe-pro phenylalanyl-proline, Val-ala valyl-alanine, Pro-leu prolyl-leucine, CA cholic acid, GDCA glycodeoxycholic acid, TDCA taurodeoxycholic acid, DCA deoxycholic acid, GCDCA glycochenodeoxycholic acid, IPTG isopropyl β-D-1-thiogalactopyranoside, THBA 2,3,4-trihydroxybutyric acid

We initially performed orthogonal partial least squares discriminant analysis (OPLS-DA) to delineate global metabolic profile alterations across three patient groups. As illustrated by the LC‒MS metabolomic data (Fig. 2a), distinct metabolic signatures were observed across the groups, indicating subtype-specific metabolic patterns. Furthermore, compared with intergroup differences, age and sex stratification had a minimal influence. GC‒MS metabolomic and lipidomic datasets demonstrated consistent alterations (Supplementary Fig. 3).

To identify differential metabolic features between the groups, we first adjusted for confounding factors (e.g., diabetes, hyperlipidemia, anxiety and depression, and the use of anti-Parkinsonian drugs) via logistic regression. Comparisons between the two groups were then performed via the Mann‒Whitney U test, and all resulting *p* values were corrected for multiple comparisons. In total, 203 significantly altered metabolites (*q* value < 0.05) were found in non-RBD-PD patients compared with Ctrl individuals (Supplementary Table 4), 154 (75.9%) of which were also dysregulated in RBD-PD patients (Fig. 2b), indicating shared core pathophysiological perturbations in the two subtypes. Among the 203 differentially expressed metabolites (DEMs), the levels of 79 metabolites (e.g., lactic acid, aspartic acid, and lysophosphatidylcholines (LPCs)) were increased, whereas the levels of 124 metabolites (e.g., mannose and acylcarnitines) were decreased in non-RBD-PD patients compared with Ctrl subjects (Fig. 2c). These dysregulated metabolites were involved in diverse chemical classes, including fatty acyls, amino acids, organic acids, carbohydrates, and bile acids, with 99.0% (201/203) exhibiting consistent directional alterations across the two PD subtypes (Fig. 2d–g, Supplementary Fig. 4).

In addition to confirming our previously reported alterations,^18^ including the depletion of fatty acids (FFAs) and acylcarnitines, the accumulation of proteolytic toxins (phenylacetylglutamine, p-cresyl glucuronide), and elevated bile acids, the present multiple-platform analysis revealed significant disruptions in energy metabolism in PD. This was characterized by the accumulation of glycolytic and tricarboxylic acid (TCA) cycle intermediates (lactic acid, pyruvic acid, malic acid, fumaric acid and α-ketoglutaric acid; Fig. 2e) and the depletion of carbohydrate metabolites (mannose, ribose, sorbose, rhamnose, etc.; Fig. 2g). Additionally, we detected a marked reduction in tryptophan-derived indole derivatives (indolelactic acid, ILA; 3-indoleacrylic acid, IAcrA; 3-indoleacetic acid, IAA; Fig. 2e) and disrupted amino acid metabolism in both PD subgroups **(**Fig. 2f**)**. These findings demonstrate conserved pathophysiological mechanisms independent of RBD status.

### Lipid profiles of PD patients

Comprehensive lipidomic profiling revealed 392 differentially expressed lipid species (DELs) in non-RBD-PD patients, of which 353 (90.1%) were similarly altered in RBD-PD patients (Fig. 2h, Supplementary Table 5). Notably, the majority of lipids were decreased in PD, with selective elevations confined to specific subclasses, including LPCs, lysophosphatidylethanolamines (LPEs), phosphatidylethanolamines (PEs), alkyl-acyl LPCs (LPC Os), and several sphingolipid species (Fig. 2i). Class-level analyses revealed increased levels of total LPC and LPC O in PD patients, whereas the total amounts of other lipid classes exhibited significant reductions (Supplementary Fig. 5a). Subsequent stratification by acyl-chain saturation revealed that the increases were confined to saturated PEs, saturated and monounsaturated LPC/LPE, and polyunsaturated LPC species (Supplementary Fig. 5b, c). Furthermore, the ratios of polyunsaturated FFAs to monounsaturated and saturated FFAs were markedly decreased in PD patients (Supplementary Fig. 5d, e). These findings suggest that aberrant lipid remodeling and impaired desaturase activity occur across PD subtypes.

### Immune-inflammatory protein alterations in PD patients

**Fig. 3:**
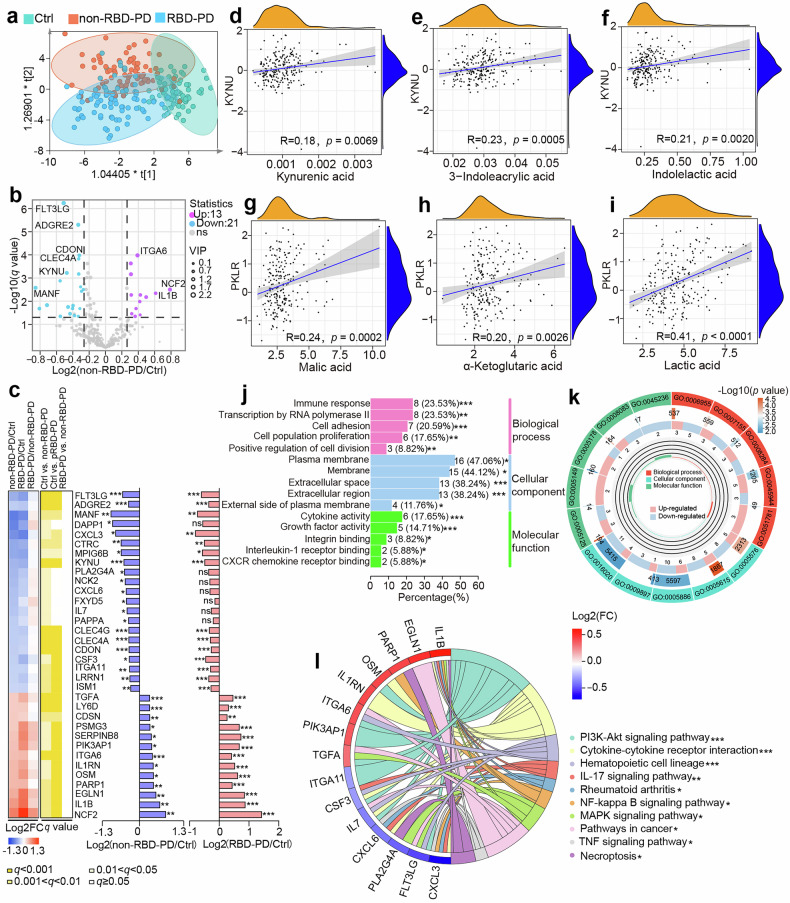
Identification of differentially expressed proteins in PD. **a** OPLS-DA model of two PD subgroups and control individuals on the basis of proteomic profile (R2Y = 0.588, F factor = 4.23, *p*-value = 6.19E-009). **b** Volcano plot visualizing differentially expressed proteins in non-RBD-PD patients compared with Ctrl subjects (*q* < 0.05, VIP > 1.0, |FC | > 1.2). **c** Heatmap visualizing the expression of 34 PD differential proteins across three groups and fold changes between non-RBD-PD patients and RBD-PD patients and healthy controls. *: 0.01 < *q* < 0.05, **: 0.001 < *q* < 0.01, ***: *q* < 0.001. The *q* value represents the FDR-adjusted *p* value for multiple hypothesis testing. The color scale in the left heatmap represents the Log2 (fold change) of each comparison, whereas the color scale in the right heatmap represents the *q*-value. **d**–**f** Correlation analysis between KYNU and metabolites (kynurenic acid, 3-indoleacrylic acid and Indolelactic acid). **g-i** Correlation analysis between PKLR and metabolites (malic acid, α-ketoglutaric acid and lactic acid). **j**, **k** Histograms and circle diagram showing the GO annotations of the DEPs. **l** Top 9 enriched KEGG pathways among the DEPs associated with PD. *: 0.01 < *p* < 0.05, **: 0.001 < *p* < 0.01, ***: *p* < 0.001. The *p*-values presented were unadjusted for multiple comparisons

Given the recognized role of neuroinflammation in PD,^21^ plasma inflammatory proteins were profiled via the Olink Explore 384 Inflammation Panel in 226 samples from Cohort 1 (Ctrl, *n* = 74; non-RBD-PD, *n* = 76; RBD-PD, *n* = 76). The OPLS-DA score plot clearly revealed separation among the groups (Fig. 3a). Following adjustment for confounders and using the criteria of *q* < 0.05, VIP > 1.0, and fold change > 1.2 (or < 0.83), we identified 34 differentially expressed proteins (DEPs) in non-RBD-PD patients (21 downregulated, 13 upregulated) (Fig. 3b, Supplementary Table 6).

Elevated expression of the proinflammatory cytokine interleukin-1β (IL-1β) and the oxidative stress-associated protein neutrophil cytosol factor 2 (NCF2) was observed in both PD subtypes (Fig. 3b, c). Moreover, we found a marked reduction in the expression of immune-related proteins, including FLT3LG (fms-related tyrosine kinase 3 ligand), ADGRE2 (adhesion G protein-coupled receptor E2), and CLEC4A (c-type lectin domain family 4 member A), which were not previously reported in PD. MANF (mesencephalic astrocyte-derived neurotrophic factor), a neuroprotective factor, was also significantly decreased in both PD subtypes. Two enzymes, KYNU (kynureninase) and PKLR (pyruvate kinase), which regulate tryptophan catabolism and glycolytic flux, also exhibited marked dysregulation. Pearson correlation analysis suggested significant positive correlations between KYNU and tryptophan metabolites (kynurenic acid, KYNA; IAcrA and ILA; Fig. 3d–f) as well as between PKLR and energy-related metabolites (malic acid, α-ketoglutaric acid and lactic acid; Fig. 3g–i).

The functional annotation revealed predominant enrichment in biological processes, including the immune response, positive regulation of transcription by RNA polymerase II and cellular adhesion (Fig. 3j, k, Supplementary Table 7). These DEPs are primarily localized in the plasma membrane, membrane, and extracellular region. KEGG pathway enrichment analysis highlighted cytokine‒cytokine receptor interactions and key signaling cascades, including the PI3K‒Akt, IL‒17, NF‒kappaB, MAPK, and TNF signaling pathways, revealing extensive activation of inflammatory and immune responses in PD (Fig. 3l, Supplementary Table 8).

### Metabolic differences between RBD-PD patients and non-RBD-PD patients

**Fig. 4:**
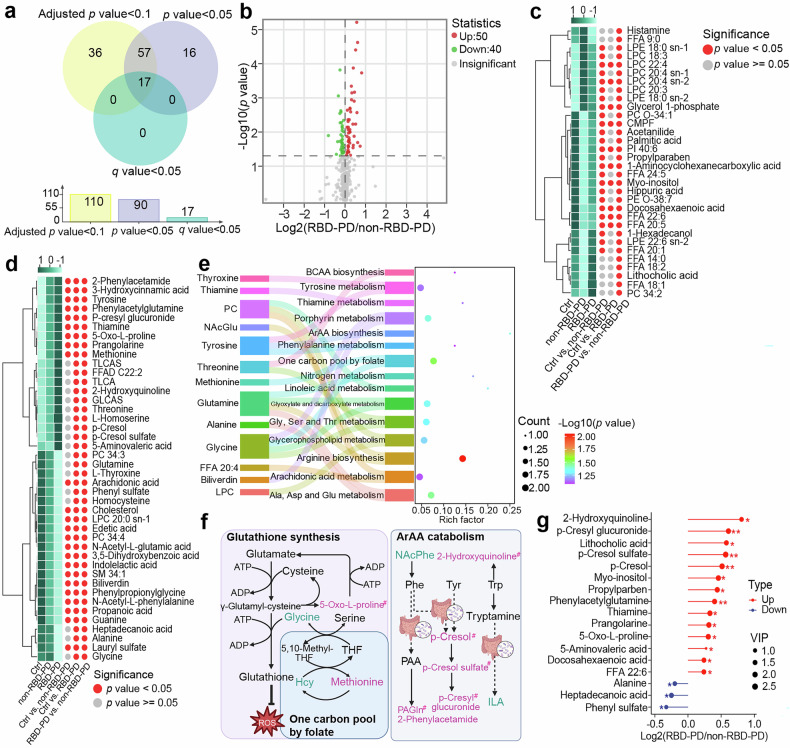
Metabolic differences between RBD-PD patients and non-RBD-PD patients. **a** Venn diagram of differentially abundant metabolites associated with RBD-PD in intergroup comparisons. Adjusted *p* values were derived from a logistic regression analysis, which was used to control for potential confounding factors. P values were calculated via the Mann‒Whitney U test, whereas *q* values represent the *p* values adjusted for multiple comparisons via the Benjamini‒Hochberg procedure. **b** Volcano plot visualizing differentially expressed metabolites in RBD-PD patients compared with non-RBD-PD patients (*p* < 0.05). **c** Heatmap of metabolites exhibiting V-shaped or inverted V-shaped concentration changes across control individuals, non-RBD-PD patients, and RBD-PD patients. **d** Heatmap of DEMs exhibiting linear level changes across control individuals, non-RBD-PD patients, and RBD-PD patients. The color scale in the heatmap represents the Z score. **e** Pathway enrichment analysis of the metabolites shown in (**d**). **f** Specific metabolic pathways associated with RBD-PD. **g** The most significantly differentially abundant metabolites in RBD-PD patients compared with non-RBD-PD patients. *: 0.01 < *q* < 0.05, **: 0.001 < *q* < 0.01, ***: *q* < 0.001. The *q* value represents the *p*-value adjusted for multiple hypothesis testing. CMPF 3-carboxy-4-methyl-5-propyl-2-furanpropionic acid, TLCAS taurolithocholic acid sulfate, TLCA taurolithocholic acid, GLCAS glycolithocholic acid sulfate, FFAD fatty acid amide, 5,10-Methylene-THF 5,10-methylenetetrahydrofolate, THF tetrahydrofolate, Hcy homocysteine, ArAA aromatic amino acid, NAcPhe N-acetyl-phenylalanine, PAGln phenylacetylglutamine, Phe phenylalanine, PAA phenylacetic acid, ILA 3-indolelactic acid, Tyr tyrosine, Trp tryptophan

To investigate subtype heterogeneity, we compared metabolic profiles between RBD-PD patients and non-RBD-PD patients. Logistic regression, adjusting for potential confounding factors, including history of diabetes, hyperlipidemia, anxiety/depression status, and anti-PD medication, yielded 110 differentially abundant metabolites (adjusted *p*-value < 0.10, Fig. 4a). The Mann‒Whitney U test identified 90 metabolites (*p*-value < 0.05, Fig. 4b), 74 of which overlapped between the two methods (Fig. 4c, d, Supplementary Table 9). Compared with non-RBD-PD patients and healthy controls, patients with RBD-PD presented more pronounced alterations in polar metabolites, including amino acids, organic acids, and secondary bile acids (Fig. 4d). In contrast, non-RBD-PD patients demonstrated greater dysregulation of lipid metabolism (Fig. 4c). To comprehensively analyze the metabolic pathways associated with RBD-PD, we focused on metabolites that exhibited progressive changes (Fig. 4d), which were involved mainly in amino acid metabolism and the one carbon pool by folate pathway (Fig. 4e). Compared with non-RBD-PD, RBD-PD exhibited significant accumulation of methionine, homoserine, threonine and 5-oxo-proline alongside a marked reduction in glycine levels. Furthermore, significant enrichment of gut microbiota-derived metabolites,^20,22^ including secondary bile acids, 2-phenylacetamide, 2-hydroxyquinoline, p-cresol, p-cresol sulfate, p-cresyl glucuronide, and phenylacetylglutamine, was detected in RBD-PD patients (Fig. 4f). Additionally, RBD-PD patients presented significant decreases in acetylated amino acids (N-acetyl-L-glutamic acid, NAcGlu; N-acetyl-L-phenylalanine, NAcPhe) and tryptophan-derived ILA levels. After correction for multiple comparisons, 17 metabolites remained significantly changed (Fig. 4g, Supplementary Fig. 6), largely representing aromatic amino acid (ArAA)-derived microbiota metabolites.

### Proteomic differences between RBD-PD patients and non-RBD-PD patients

**Fig. 5:**
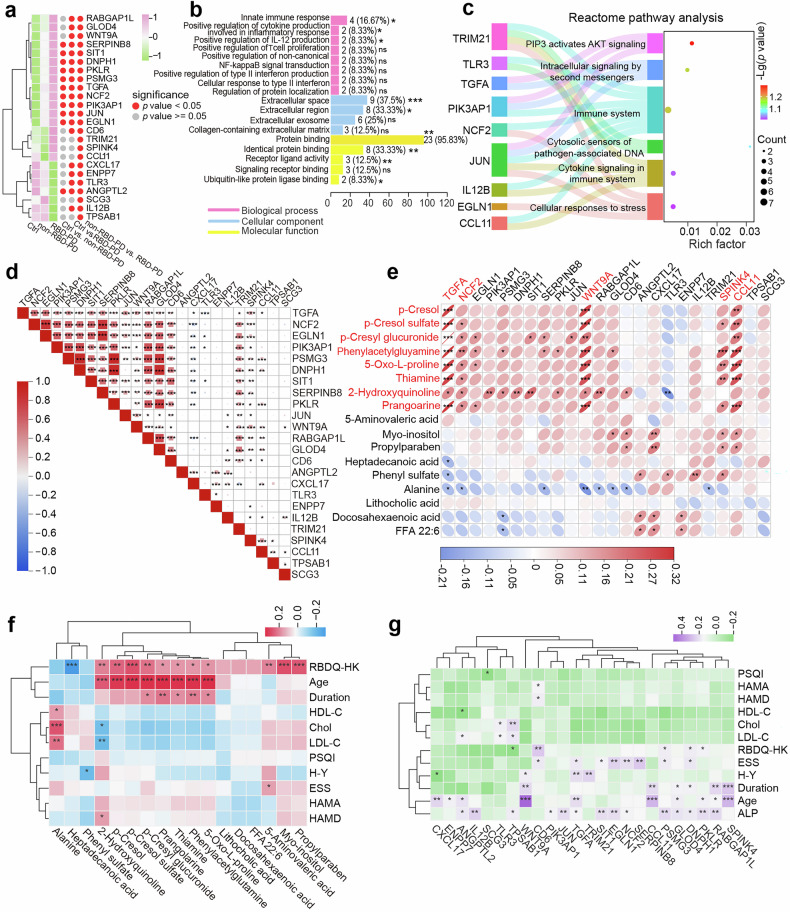
Proteomic differences between RBD-PD patients and non-RBD-PD patients. **a** Relative concentration changes across three groups and statistical significance in group comparisons for DEPs between PD patients with and without RBD. The color scale in the heatmap represents the Z score. **b** Histograms showing GO annotations for RBD-PD-related DEPs. *: 0.01 < *p* < 0.05, **: 0.001 < *p* < 0.01, ***: *p* < 0.001. The *p*-values presented were unadjusted for multiple comparisons. **c** Reactome pathway enrichment analysis for the 24 DEPs identified in RBD-PD. **d** Pearson correlation analysis of the 24 RBD-PD-related DEPs. The color scale in the heatmap represents the correlation coefficient. **e** Pearson correlation analysis between 24 DEPs and 17 DEMs in RBD-PD patients. **f** Pearson correlation analysis between the top 17 DEMs of RBD-PD patients and the clinical characteristics of patients. **g** Pearson correlations between 24 DEPs associated with RBD-PD and the clinical characteristics of patients. *: 0.01 < *p* < 0.05, **: 0.001 < *p* < 0.01, ***: *p* < 0.001. H-Y Hoehn-Yahr, PSQI Pittsburgh sleep quality index, RBDQ-HK REM sleep behavior disorder questionnaire - Hong Kong, ESS Epworth sleepiness scale, HAMA Hamilton Anxiety Rating Scale, HAMD Hamilton Depression Rating Scale, Chol total cholesterol, LDL-C low-density lipoprotein cholesterol, ALP alkaline phosphatase

Following adjustment for confounders and univariate analysis, we identified 24 protein candidates differentiating RBD-PD patients from non-RBD-PD patients (Fig. 5a). However, none of these proteins remained statistically significant after correction for multiple comparisons (Supplementary Table 10). Given the exploratory nature of the study, we proceeded with Gene Ontology (GO) enrichment analysis, which suggested the involvement of the innate immune response, positive regulation of cytokine production involved in the inflammatory response, and positive regulation of IL-12 production. These DEPs predominantly localize to the extracellular space and primarily engage in protein binding activities (Fig. 5b, Supplementary Table 11). Reactome pathway analysis demonstrated significant enrichment of key pathways, including PIP3 activation of AKT signaling, the immune system, cytokine signaling in the immune system, and cellular responses to stress (Fig. 5c, Supplementary Table 12). Additionally, Pearson correlation analysis of these proteins revealed strong correlations, particularly among TGFA, NCF2, EGLN1, PIK3AP1, PSMG3, DNPH1, SIT1, SERPINB8, and PKLR (Fig. 5d).

### Correlations between proteins and metabolites

To further explore the interplay between proteomic and metabolomic alterations in RBD-PD patients, we correlated these DEPs with the most significantly altered metabolites shown in Fig. 4g via Pearson correlation analysis. We found that the levels of gut microbiota-derived metabolites (p-cresol, p-cresol sulfate, p-cresyl glucuronide, and phenylacetylglutamine) were highly correlated with the expression of key proteins, including the neuroinflammation-related marker CCL11 (eotaxin), the intestinal inflammation-associated protein SPINK4 (serine protease inhibitor Kazal-type 4), the oxidative stress-linked protein NCF2, and the signaling proteins TGFA (protransforming growth factor alpha) and WNT9A (protein Wnt-9a). Conversely, alanine was significantly negatively correlated with these proteins (Fig. 5e).

### Correlations between molecular signatures and clinical characteristics

We further investigated the correlations between RBD-PD-related metabolites/proteins and the clinical characteristics of patients. We found that the levels of heptadecanoic acid were significantly negatively correlated with the RBDQ-HK scores. Conversely, the levels of 2-hydroxyquinoline, p-cresol, p-cresol sulfate, p-cresyl glucuronide, phenylacetylglutamine and 5-oxo-L-proline were significantly positively correlated with both the RBDQ-HK score and age (Fig. 5f).

Similar to the metabolic findings, the expression levels of proteins, including SPINK4, CCL11, and WNT9A, also demonstrated significant correlations with disease duration and age (Fig. 5g). Furthermore, the expression of proteins, including CD6 (T-cell differentiation antigen CD6) and PKLR, was positively correlated with the RBDQ-HK score, indicating their potential association with RBD-related comorbidities. Notably, proteins such as NCF2 and SERPINB8 (serpin B8) were positively correlated with ESS scores, indicating their potential roles in the pathogenesis of sleep disturbances.

### Biomarker identification for PD subtype discrimination

**Fig. 6:**
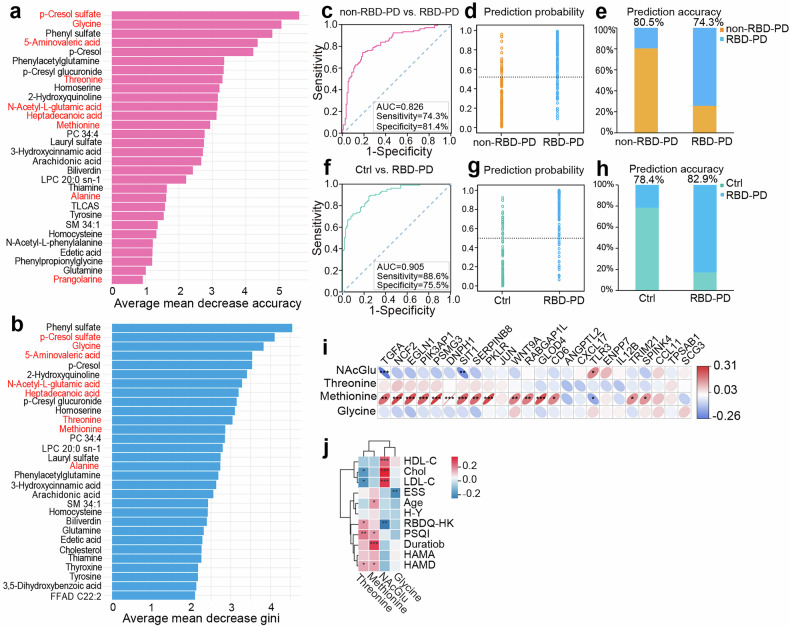
Establishment of a biomarker model to distinguish RBD-PD patients from non-RBD-PD patients. **a** Mean decrease in accuracy of the top 30 signatures identified by random forest. This metric represents the magnitude of reduction in prediction accuracy when a variable’s values are permuted; a larger decrease indicates greater importance of that variable. **b** Mean decrease gini of the top 30 signatures identified by random forest. Variable importance is evaluated by measuring the total decrease in node impurity (calculated by the Gini index) resulting from splits over a given variable. A higher value indicates greater importance. **c** ROC curve evaluating the performance of the biomarker model in distinguishing between two clinical subtypes of PD. **d**, **e** Prediction probability and prediction accuracy of the classification model in distinguishing between two clinical subtypes. **f** ROC curve evaluating the performance of the classification model in distinguishing RBD-PD patients from Ctrl individuals. **g**, **h** Prediction probability and prediction accuracy of the classification model in distinguishing RBD-PD patients and Ctrl subjects. **i** Pearson correlation between the levels of metabolites and 24 DEPs. **j** Pearson correlation of the levels of metabolites and clinical parameters. *: 0.01 < *p* < 0.05, **: 0.001 < *p* < 0.01, ***: *p* < 0.001. TLCAS taurolithocholic acid sulfate, FFAD fatty acid amide, NAcGlu N-acetyl-glutamic acid

We adopted a two-stage pipeline comprising random forest (RF) and binary logistic regression (BLR) to identify candidate biomarkers for PD subtype discrimination. Initially, the 42 metabolites that exhibited progressive changes in RBD-PD (Fig. 4d) were used to construct an RF classification model using 500 decision trees. The importance of each feature in classifying the two subtypes was quantified via the mean decrease in accuracy (MDA) and mean decrease in Gini (MDG) metrics from 999 runs. The top 30 metabolites ranked by RF were subsequently identified (Fig. 6a, b) and subjected to BLR analysis. Using a forward stepwise selection algorithm, we identified an optimal predictive model consisting of a panel of 9 metabolites, including p-cresol sulfate, glycine, 5-aminovaleric acid, threonine, N-acetyl-L-glutamic acid, heptadecanoic acid, methionine, alanine and prangolarine **(**Supplementary Fig. 7, Supplementary Table 13). Receiver operating characteristic (ROC) curve analysis of the panel yielded an area under the curve (AUC) of 0.826 for separating RBD-PD patients from non-RBD-PD patients, with 74.3% sensitivity and 81.4% specificity. The predictive accuracies were 80.5% and 74.3% for the two subtypes, respectively (Fig. 6c–e). Furthermore, we evaluated the panel’s performance in distinguishing RBD-PD patients from Ctrl, which resulted in an AUC of 0.905, with a sensitivity of 88.6% and a specificity of 75.5%. The predictive accuracies for the Ctrl and RBD-PD patients were 78.4% and 82.9%, respectively (Fig. 6f–h, Supplementary Table 14).

To obtain a robust and unbiased estimate of the model’s predictive performance, we performed an iterative leave-group-out cross-validation analysis. Following 1000 cross-validation runs, the biomarker panel achieved a mean AUC of 0.787, with a mean sensitivity of 74.0% and specificity of 69.9% for distinguishing PD subtypes, and a mean AUC of 0.873 (76.7% sensitivity, 79.7% specificity) for distinguishing RBD-PD patients from Ctrl. Moreover, six metabolites in the panel (excluding 5-aminovaleric acid, threonine and glycine) were significantly correlated with the RBD-PD-related DEPs (Fig. 5e, Fig. 6i), and eight (excluding alanine) were significantly correlated with the clinical sleep parameters, particularly the RBDQ-HK scores (Fig. 5f, Fig. 6j).

### Validation in an independent cohort

**Fig. 7:**
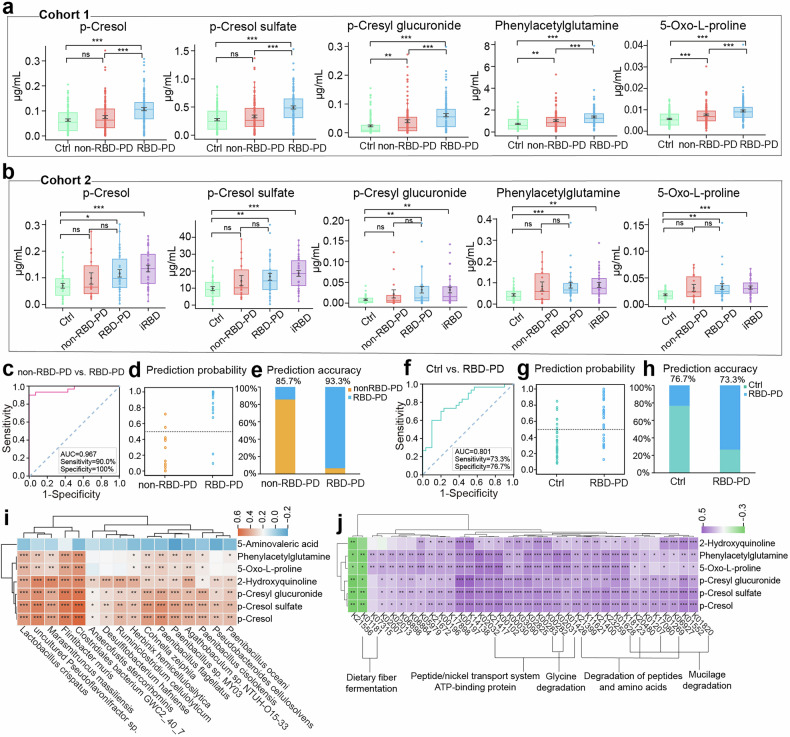
Biomarker validation and correlations with the gut microbiota. **a**, **b** Box-and-whisker plots showing the relative concentrations of p-cresol, p-cresol sulfate, p-cresyl glucuronide, phenylacetylglutamine, and 5-oxo-L-proline across groups in Cohorts 1 and 2. **c** ROC curve evaluating the performance of the biomarker model in distinguishing between two clinical subtypes in Cohort 2. **d**, **e** Prediction probability and prediction accuracy of the classification model in distinguishing between two clinical subtypes in Cohort 2. **f** ROC curve evaluating the performance of the classification model in distinguishing RBD-PD from Ctrl in Cohort 2. **g**, **h** Prediction probability and prediction accuracy of the classification model in distinguishing RBD-PD from Ctrl in Cohort 2. **i** Spearman correlation analysis between metabolite levels and the abundance of the top 16 significantly altered microbial species. **j** Spearman correlation analysis between metabolite levels and the abundance of the top 38 significantly altered functional genes. *: 0.01 < *p* < 0.05, **: 0.001 < *p* < 0.01, ***: *p* < 0.001

In an independent clinical cohort (Cohort 2) comprising vPSG-confirmed patients with non-RBD-PD, RBD-PD, or iRBD and healthy controls, 10 of the 17 top metabolites identified from Cohort 1 exhibited consistent trends (Supplementary Table 15). Notably, 5 of these metabolites, including p-cresol, p-cresol sulfate, p-cresyl glucuronide, phenylacetylglutamine and 5-oxo-L-proline, were significantly increased in both the RBD-PD and iRBD patients (Fig. 7a, b).

We then evaluated the 9-metabolite model in Cohort 2, which yielded excellent performance in distinguishing RBD-PD patients from non-RBD-PD patients, achieving an AUC of 0.967 (90.0% sensitivity, 100% specificity), with predictive accuracies of 85.7% (non-RBD-PD patients) and 93.3% (RBD-PD patients) (Fig. 7c–e, Supplementary Table 16). When distinguishing RBD-PD patients from controls, the model yielded an AUC of 0.801 (73.3% sensitivity, 76.7% specificity), with predictive accuracies of 76.7% (Ctrl) and 73.3% (RBD-PD) (Fig. 7f–h, Supplementary Table 17).

### Associations between gut microbial dysbiosis and metabolic profiles

Additionally, we performed metagenomic sequencing in 73 participants from Cohort 2. We first identified the top 16 microbial species that were significantly differentially abundant in both the RBD-PD and iRBD groups **(**Supplementary Table 18, details in the Methods section). Correlation analysis revealed that the relative abundances of these microbial species were significantly correlated with the levels of key RBD-PD metabolites. This was particularly evident for species such as *Lactobacillus crispatus*, *uncultured Pseudoflavonifractor sp*., *Marasmitruncus massiliensis*, *Flintibacter muris*, *Clostridiales bacterium GWC2_40_7*, and *Paenibacillus flagellatus* (Fig. 7i). Furthermore, we identified the top 38 functional genes (KEGG orthologs, KOs) that were significantly dysregulated in both the RBD-PD and iRBD groups (Supplementary Table 19, details in the Methods section). Notably, these key metabolites were significantly negatively correlated with K21556 (*malR*, CRP/FNR family transcriptional regulator) and K01181 (*xynA*, endo-1,4-beta-xylanase), which were significantly downregulated in both RBD-PD and iRBD. In contrast, significant positive correlations were detected between metabolites and functional genes involved in ATP-binding cassette transport (K02031, K02032), peptide and amino acid degradation (K01426, K18123, and K01269), and mucus degradation (K01628, K01207, and K01820; Fig. 7j).

## Discussion

Although multiomics technologies, including metabolomics, proteomics and microbiomics, have substantially advanced our understanding of PD, several challenges remain. Differences in analytical platforms lead to inconsistent molecular coverage across studies, leaving many PD-associated metabolites, proteins and their interactions unresolved. Moreover, the differential molecular signatures distinguishing PD patients with and without RBD, as well as their roles in subtype-specific pathophysiology, have not been fully elucidated.

In this study, we integrated multiplatform metabolomics and proteomics to profile plasma molecular changes in patients with RBD-PD, non-RBD-PD, and healthy controls. This integrative approach revealed marked metabolic reprogramming and dysregulated immunoinflammatory responses in PD. Furthermore, we identified subtype-specific molecular alterations, most notably the accumulation of gut-derived proteolytic toxins in RBD-PD patients. On the basis of these signatures, we constructed a robust biomarker panel capable of stratifying PD subtypes. These findings provide a framework for unraveling PD heterogeneity and highlighting biologically relevant molecular differences across subtypes.

**Fig. 8:**
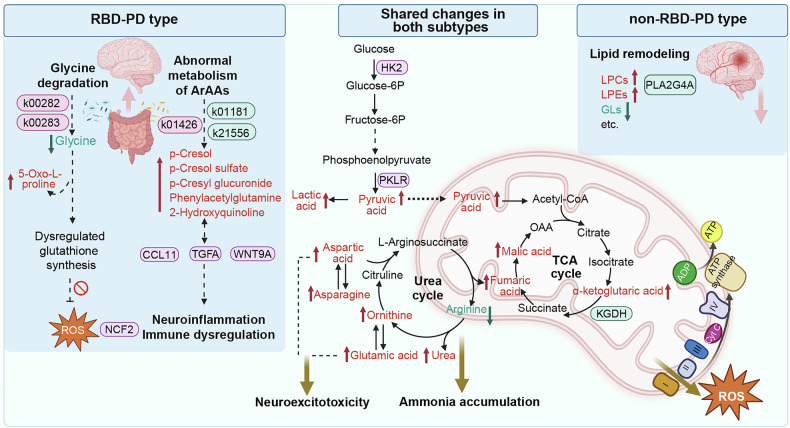
Schematic illustration of metabolic pathway dysregulation in PD subtypes. Both RBD-PD patients and non-RBD-PD patients exhibit substantial shared alterations at the metabolic and proteomic levels, which are closely associated with mitochondrial dysfunction and neuroinflammatory processes. The significant downregulation of key metabolic enzymes and the accumulation of metabolites in the TCA cycle suggest impaired TCA cycle flux in PD. Significant increases in urea and urea cycle metabolites may directly induce systemic ammonia accumulation, which subsequently inhibits KGDH activity. Additionally, this study demonstrated that RBD-PD patients exhibit distinctive metabolic features, including impaired glutathione synthesis and gut microbiota dysbiosis-derived accumulation of toxic ArAA degradation products, as well as exacerbated immune dysfunction and inflammatory activation. In contrast, non-RBD-PD patients demonstrated more substantial dysregulation of lipid metabolism. ArAA aromatic amino acids, Glucose-6P glucose 6-phosphate, Fructose-6P fructose 6-phosphate; OAA oxaloacetate. Figure 8 was generated via BioRender (https://www.biorender.com)

Both RBD-PD and non-RBD-PD patients presented substantial metabolic and proteomic abnormalities (Fig. 8). These common changes are closely associated with their overlapping pathophysiological features, including α-synuclein aggregation, mitochondrial dysfunction, and neuroinflammation. Mitochondrial dysfunction is well recognized in PD,^23^ and brain glucose hypometabolism has been reported, even in the early stages of PD.^24–26^ We found that the levels of metabolites in the TCA cycle were significantly increased in both PD subtypes (Fig. 2e). This finding is consistent with the previously reported downregulation of key metabolic enzymes in the TCA cycle, including pyruvate dehydrogenase (PDH), isocitrate dehydrogenase 3α (IDH3A), citrate synthase (CS), and α-ketoglutarate dehydrogenase (KGDH), in multiple brain regions of PD patients.^27^ These findings suggest that impaired TCA cycle flux in PD patients might pathologically contribute to the accumulation of upstream intermediates.

Conversely, prior work has demonstrated increased expression of hexokinases (HK2 and HK3) in the substantia nigra pars compacta of both PD patients and the MPTP (1-methyl-4-phenyl-1,2,3,6-tetrahydropyridine)-induced mouse model,^26,28^ supporting a metabolic shift from the TCA cycle to glycolysis in PD. This Warburg-like shift in metabolism can be triggered by the loss of mitochondrial complex I (MCI) function resulting from *Ndufs2* deletion in dopaminergic neurons.^29^ While this adaptation transiently sustains neuronal viability, it can ultimately trigger a progressive loss of the dopaminergic phenotype.^29^ Furthermore, the accumulation of lactic acid and TCA cycle intermediates can instigate a feedforward cascade that exacerbates neurodegenerative progression. It has been reported that mitochondrial fumaric acid inhibits PINK1-Parkin-mediated mitophagy through the succination of specific cysteine residues, which disrupts Parkin function and induces PD-related phenotypes.^30^ Notably, targeted modulation of TCA cycle enzymes such as KGDH has been demonstrated to reduce lipid peroxidation and suppress α-synuclein phosphorylation,^23^ indicating the therapeutic potential of metabolic interventions in PD.

The coupling between the TCA cycle and the urea cycle is critically important for ammonia detoxification and energy homeostasis regulation.^31^ Our findings revealed significant increases in the levels of urea and urea cycle metabolites (e.g., ornithine, aspartic acid, asparagine and glutamic acid; Fig. 2f, Supplementary Fig. 4c). This urea cycle dysfunction can directly induce systemic ammonia accumulation, which subsequently inhibits KGDH activity.^32^ Moreover, impairment of the TCA cycle leads to ATP depletion, further compromising the ammonia detoxification capacity and establishing a vicious cycle.^33^ Furthermore, activated microglia generate excessive nitric oxide, which can suppress MCI activity and compete with urea cycle-derived arginine.^34^ This competition may disrupt polyamine metabolism, thereby accelerating α-synuclein aggregation.^35–37^ Additionally, the pathological accumulation of excitatory amino acids (glutamic acid and aspartic acid) resulting from urea cycle impairment leads to aberrant overactivation of NMDA and AMPA receptors, triggering dysregulated calcium influx.^38^ The resulting calcium dyshomeostasis further contributes to mitochondrial dysfunction, excessive reactive oxygen species (ROS) production, and oxidative stress, collectively driving the degeneration and death of dopaminergic neurons.^38^

Lipid metabolism dysregulation was another prominent shared feature in both PD subtypes observed in this study. Notably, most lipid species exhibited significant reductions across both subtypes, whereas the majority of LPCs and LPEs were markedly elevated (Fig. 2i, Supplementary Fig. 5a–c), indicating substantial lipid remodeling. A recent study reported that LPC can disrupt the autophagolysosomal pathway and lysosomal acidification by activating cleaved caspase-3 via the orphan receptor GPR35-ERK signaling pathway, further exacerbating the accumulation of toxic α-synuclein.^39^ Given that lipids constitute fundamental structural components of biological membranes, lipid remodeling in PD can profoundly affect membrane architecture, functionality, and fluidity. Consistently, GO functional annotation demonstrated that the majority of DEPs associated with PD are localized to membrane compartments. In particular, proteins, including PIK3AP1 and PLA2G4A (Fig. 3c), which are involved in phospholipid metabolism, membrane assembly, and intracellular membrane trafficking, were reported to be differentially expressed in PD for the first time in this study. Although direct evidence is lacking, a study reported that loss of function of PLA2G6, a homolog of PLA2G4A, can impair synaptic transmission and lead to neurodegeneration.^40^ PIK3AP1 serves as a negative regulator of the PI3K-AKT pathway.^41^ This unique transmembrane protein can inhibit PI3K signaling by inducing proteasomal degradation of PIK3R3.^41^ The PI3K-AKT pathway is recognized as neuroprotective against the development and progression of PD and is involved in multiple cellular processes, such as ERK signaling, oxidative stress regulation, endoplasmic reticulum stress, calcium homeostasis, and autophagy.^42,43^ Multiple natural products, such as curcumin and quercetin, have exhibited neuroprotective effects by activating the PI3K-AKT pathway.^42,44^ Therefore, pharmacological targeting of PIK3AP1 to restore PI3K-AKT signaling might represent a novel therapeutic strategy.

In addition to the shared alterations, we identified distinct molecular differences between RBD-PD and non-RBD-PD. The non-RBD-PD subtype exhibited more pronounced lipidomic perturbations, characterized by significant elevations in LPCs, along with marked reductions in phospholipids and polyunsaturated FFAs. Conversely, the RBD-PD subtype demonstrated an exacerbated accumulation of gut-derived toxic metabolites and impairment of glutathione (GSH) synthesis (Fig. 8).

Substantial evidence indicates marked GSH depletion in PD brains, particularly in the substantia nigra pars compacta, a pathological change notably preceding clinical symptom manifestation.^45,46^ Our metabolomic profiling revealed decreased levels of glycine, a critical precursor for GSH synthesis, in PD patients, especially in those with RBD-PD. Glycine deficiency and the subsequent decrease in GSH production lead to the accumulation of γ-glutamyl-cysteine.^47^ This intermediate is subsequently shunted by γ-glutamyl-cyclotransferase, leading to increased production of 5-oxo-L-proline, which is a hallmark of impaired de novo GSH biosynthesis.^47^ Notably, our metagenomic analysis revealed that gut dysbiosis in patients with RBD-PD and iRBD leads to the upregulation of K00282 (*gcvPA*, glycine dehydrogenase subunit 1) and K00283 (*gcvPB*, glycine dehydrogenase subunit 2), two functional genes involved in the glycine degradation pathway (Fig. 7j). The abundance of these two functional genes was significantly correlated with the 5-oxo-L-proline level. These findings suggest that the functional dysbiosis of the gut microbiome also contributes to the observed impairment in GSH synthesis. Since GSH is the primary endogenous antioxidant and pivotal scavenger of ROS, its depletion triggers a cascade of exacerbated oxidative stress, which induces progressive degeneration of dopaminergic neurons.^48^ This finding is in line with the elevated expression of NCF2 observed in RBD-PD patients. NCF2 serves as an essential component of the NADPH oxidase complex, which critically regulates the generation of ROS.^49^ The present study revealed for the first time that the expression of NCF2 is significantly increased in the RBD-PD subtype. Although direct evidence remains unavailable, a previous study reported that the expression of the *NCF2* gene is downregulated in PD patients but upregulated in patients with inflammatory bowel disease.^49^ The overexpression of NCF2 in RBD-PD patients may therefore be closely related to the gastrointestinal dysfunction observed in this subtype.

Another key finding was the remarkable elevation of gut microbiota-derived metabolites in RBD-PD, such as the tyrosine-derived phenolic compound p-cresol and its conjugates (p-cresol sulfate and p-cresyl glucuronide), as well as phenylalanine-derived phenylacetylglutamine. These metabolites, produced by gut microbial fermentation of ArAAs, were increased in both RBD-PD and iRBD patients, suggesting that these metabolic alterations may be innate to this subtype rather than merely a consequence of PD disease progression. Previous studies have shown that the members of the order *Clostridiales*, particularly *Clostridiaceae* and *Ruminococcaceae*, are primary fermenters of ArAAs and producers of p-cresol.^50,51^ We found that the abundances of *Clostridiales bacterium GWC2_40_7* and *Ruminiclostridium cellulolyticum* (both belonging to the order *Clostridiales)*, as well as *Flintibacter muris*, were significantly increased in both RBD-PD patients and iRBD patients. This enrichment may contribute to the increase in p-cresol and its hepatic conjugated forms. P-cresol and its metabolites, particularly p-cresol sulfate, are highly cytotoxic, inducing oxidative stress, mitochondrial dysfunction, neuroinflammation, and synaptic disruption.^52^ Recent studies have demonstrated that p-cresol disrupts the integrity of the blood–brain barrier (BBB) by activating epidermal growth factor receptor (EGFR) signaling and promoting matrix metalloproteinase release, thereby inducing extracellular matrix degradation, cytoskeletal changes, and weakened cell junctions, ultimately leading to increased BBB permeability.^52,53^ Critically, the elevations in p-cresol and its metabolites were strongly linked to increased levels of the immune-inflammatory proteins TGFA, NCF2, WNT9A, and CCL11 (Fig. 5e).

Our metagenomic functional analysis revealed microbial shifts that drive the accumulation of these toxic metabolites. The gut microbiota of both RBD-PD patients and iRBD patients demonstrated a functional transition away from the effective utilization and fermentation of dietary fiber and toward the degradation of proteins and amino acids. K21556 and K01181 are key genes encoding enzymes that regulate the polysaccharide utilization system for producing short-chain FFAs.^54^ The depletion of these genes suggests an impairment in dietary fiber fermentation in both RBD-PD patients and iRBD patients. Consistent with our findings, previous studies reported a significant enrichment of genera such as *Akkermansia, Collinsella*, and *Desulfovibrio*, as well as a significant depletion of butyrate-producing bacteria (e.g., *Butyricicoccus*, *Faecalibacterium*, and *Lachnospira*) in both iRBD patients and their first-degree relatives.^1^ These alterations are highly consistent with those observed in early PD patients.^1,55^

Conversely, genes involved in protein transport (K02031, *ddpD*, and K02032, *ddpF*, peptide/nickel transport system ATP-binding proteins) and phenylalanine catabolism (K01269, *yhfE*, aminopeptidase; K01426, *amiE*, amidase)^56^ were significantly upregulated, providing evidence for enhanced proteolysis and the generation of toxic products. Additionally, we found a significant enrichment of functional genes (K01207, *nagZ*, beta-N-acetylhexosaminidase; K01628, *fucA*, L-fuculose-phosphate aldolase) involved in amino sugar and fucose metabolism in both RBD-PD patients and iRBD patients. These sugars are major components of the intestinal mucus layer (mucin).^57,58^ The enrichment of these genes suggests accelerated mucin degradation, further compromising epithelial barrier integrity in RBD-PD and iRBD patients.

In summary, our multiomics analyses revealed convergent metabolic shifts in both the RBD-PD and non-RBD-PD subtypes, characterized by inhibited TCA cycle flux, urea cycle impairment, and substantial lipid remodeling. These metabolic aberrations collectively may exacerbate mitochondrial dysfunction, oxidative stress, neuroinflammation, and altered immune responses. We also demonstrated a more severe accumulation of gut-derived toxic proteolytic metabolites in RBD-PD patients, with these alterations already present in the prodromal stage, iRBD. The integration of metagenomics provides evidence that gut dysbiosis induces a functional shift away from dietary fiber fermentation toward protein and mucin degradation, which might contribute to these metabolic abnormalities. This interplay between the microbiome and host metabolism jointly promotes dopaminergic neurodegeneration and accelerates pathological progression. These findings expand our understanding of the heterogeneity of PD and reveal potential biological pathways for therapeutic intervention.

Nonetheless, several limitations remain. First, although we adjusted for key confounders such as hyperglycemia, hyperlipidemia, and antiparkinsonian medication, other covariates that may impact the molecular profiles (e.g., smoking status, dietary habits, and environmental factors) were not comprehensively controlled. Second, the diagnosis of RBD-PD in Cohort 1 was questionnaire-based, which may not fully match the vPSG-confirmed diagnoses. To address this, we validated the key RBD-PD-associated metabolites in an independent vPSG-confirmed cohort (Cohort 2), despite the relatively limited sample size. Given that ethnic, genetic, environmental, and healthcare system differences may impact multiomics findings, future validation in large-scale, multicenter cohorts is warranted. Third, our proteomic analysis was restricted to inflammation- and immune-related proteins. Consequently, investigations into potential changes in other protein classes, such as those involved in neuronal and synaptic function, mitochondrial function, or structural proteins, are limited. Finally, this cross-sectional design limits causal inference, and longitudinal investigations are needed to determine the temporal evolution of these molecular signatures and assess their predictive value for disease progression or phenotypic conversion.

## Materials and methods

### Subject recruitment

This study was based on two independent cohorts comprising the discovery phase (Cohort 1) and the validation phase (Cohort 2). Participants in Cohort 1 were recruited from July 2016 to May 2024 at the First Affiliated Hospital of Dalian Medical University, with ethical approval from the hospital Ethics Committee (reference No. LCKY2014-29). Participants in Cohort 2 were recruited from March 2023 to July 2025 at the Affiliated Brain Hospital of Guangzhou Medical University (ethics approval reference No. 2022.023; cohort register reference No. ChiCTR2400092208). This study was conducted in accordance with the Declaration of Helsinki. All participants provided written informed consent.

The inclusion criteria for PD patients in both cohorts included the following: (1) diagnosis confirmed by two independent neurologists according to the Movement Disorder Society Clinical Diagnostic Criteria for PD;^59^ (2) exclusion of Parkinsonian syndromes or other similar disorders; (3) exclusion of comorbidities, including severe diabetes and hypertension; and (4) exclusion of patients receiving antibiotic treatment within one month prior to enrollment.

To define RBD, we used the Rapid Eye Movement Sleep Behavior Disorder Screening Questionnaire-Hong Kong (RBDQ-HK) in Cohort 1. This is a validated tool to diagnose clinically probable RBD with a cutoff score above 18 (82.2% sensitivity, 86.9% specificity).^60–62^ In Cohort 2, RBD diagnosis was confirmed according to the International Classification of Sleep Disorders (3rd edition) on the basis of a history of dream-enactment behavior and increased electromyographic activity in submental and limb muscles determined by vPSG during REM sleep.^63^ The participants were subjected to comprehensive neurological assessments via standardized protocols. The two cohorts included several measurements, including the Hoehn–Yahr staging for evaluating motor function severity,^64^ the Epworth Sleepiness Scale (ESS) for assessing daytime sleepiness, and the RBDQ-HK for quantifying RBD severity. Specifically, in Cohort 1, the Pittsburgh Sleep Quality Index (PSQI) was used to measure overall sleep quality.^65^

### Plasma sample collection

Following overnight fasting, peripheral venous blood was collected into an EDTA-anticoagulated tube. The samples were processed using a two-step centrifugation protocol: first, they were centrifuged at 1000 × g for 10 min to separate the cellular components, followed by centrifugation of the supernatant at 2000 × g for 5 min to obtain platelet-poor plasma. The plasma was aliquoted and stored at −80 °C until analysis.

### Metabolomics and lipidomics analysis

For metabolomic profiling, the metabolite extraction protocol was described in our previous work.^18^ For GC‒MS analysis, the metabolite extracts underwent a two-step derivatization process involving oximation followed by silylation prior to analysis.^66^ For lipidomics analysis, a biphasic extraction system utilizing methyl tert-butyl ether/methanol/water was implemented to extract lipid species.

Three analytical platforms were combined to maximize metabolome coverage: LC‒MS in positive ionization mode (ESI+) targets basic and positively charged metabolites, LC‒MS in negative ionization mode (ESI-) specifically detects acidic and negatively charged metabolites, and GC‒MS profiles low-polarity or volatile metabolites that are incompatible with the reversed-phase LC, thereby ensuring comprehensive detection of chemically diverse metabolite classes. The detailed chromatographic gradients and mass spectrometric acquisition parameters for each analytical platform are described in our previous studies.^18,66^

### Metabolite identification

For LC‒MS analysis, data were acquired in information-dependent acquisition mode to comprehensively collect exact mass (*m/z)*, retention time (t_R_), and secondary MS/MS spectra at low (15 eV), medium (30 eV), and high (45 eV) collision energies. Metabolite identification was achieved by matching *m/z* (±10 ppm), t_R_ (±0.15 min), and MS/MS spectral data against both the MS-DIAL database and an in-house reference library (OSI/SMMS) established using authentic chemical standards.^67,68^ For GC‒MS analysis, metabolite identification followed our prior strategy, involving preliminary structural assignment via database searches (NIST 11, Mainlib, replib, Fiehn, etc.) and definitive confirmation through retention index matching and spectral verification using authentic standards.^66^ Detailed structural annotations for all the detected metabolites are provided in Supplementary Tables 1–3.

### Raw data processing and normalization

To ensure accurate quantification, the chromatographic peaks for all identified metabolites in LC‒MS-based metabolomics were manually integrated via SCIEX OS (Ver.2.1.6.59781) software. In total, 241 metabolites were identified in the ESI+ mode, and 189 metabolites were identified in the ESI- mode.^18^ For the GC‒MS data, 190 ion features were annotated with definite metabolite information.^66^ A quantitative method based on specific retention times and characteristic ions for these identified metabolites was established via postrun analysis software (Shimadzu, Kyoto, Japan). Peak area integration across all samples was then performed via GC‒MS browser software (Shimadzu, Kyoto, Japan).^66^ Following dereplication, a total of 455 unique metabolites were retained from the LC‒MS and GC‒MS analyses. All lipidomics raw data files were processed through the MS-DIAL software pipeline, which encompasses chromatographic peak alignment, feature extraction, and integration to acquire peak areas for each lipid species.^66^ The analysis yielded 802 lipids identified in ESI+ mode and 348 lipids identified in ESI- mode. After removing duplicate lipid species, 771 unique lipid species were ultimately characterized and used for statistical analysis.

Missing values in the datasets, which largely result from biological or technical reasons (e.g., concentrations below the limit of detection or biological absence), were handled in two steps. First, features with missing value frequencies greater than 20% in all three sample groups were removed. All remaining missing values in the filtered datasets were subsequently imputed with zero prior to analysis.^69^ To enhance comparability across analytical batches and platforms, raw peak areas were calibrated using a panel of stable isotope-labeled ISs that had been spiked into the extraction solvents (detailed in the Supplementary Materials). For the GC‒MS data, an additional signal drift correction via the local mean signal correction algorithm (LoMec, which is based on the mean value of two adjacent QC samples) was applied to the raw data prior to IS calibration.

### Proteomic profiling and data processing

Proteomic analysis was performed via the proximity extension assay technique (Olink Bioscience AB). We selected the Olink Explore 384 Inflammation Panel, which comprises 363 oligonucleotide-labeled antibody probe pairs designed to specifically bind inflammation-associated target proteins in samples, enabling subsequent detection and quantification via polymerase chain reaction amplification coupled with next-generation sequencing (NGS). NGS was performed on an Illumina NovaSeq 6000 platform. The incubation, extension, and detection steps of the Olink experiment were performed by Sinotech Genomics Co., Ltd. (Shanghai, China).

The raw NGS data were processed via Olink bcl2count™ software to generate counts .csv files. The resulting count files were imported into Olink NPX Manager to generate normalized protein expression (NPX) data, which were used for downstream bioinformatic analysis pipelines. The calculation of the NPX values involves two steps. First, the assay counts for each sample are normalized to the known standard (extension control), followed by a log2 transformation. The second step is intensity normalization, which is achieved by subtracting the median value of all samples (excluding the control strip) from each sample’s log2-transformed value. The NPX generation process is mathematically defined by the following equations, where *i* indicates a specific assay, *j* indicates a sample, and ExtNPX represents the extension-normalized NPX value.

$${{\rm{ExtNPX}}}_{{\rm{i}},{\rm{j}}}=\log 2({\rm{counts}}\left({{\rm{sample}}}_{{\rm{j}}}{{\rm{Assay}}}_{{\rm{i}}}\right)/{\rm{counts}}\left({\rm{extension}}\,{{\rm{control}}}_{{\rm{j}}}\right)$$

$${\mathrm{NPX}}_{{\rm{i}},{\rm{j}}}={\mathrm{ExtNPX}}_{{\rm{i}},{\rm{j}}}-\mathrm{median}\left(\mathrm{ExtNPX}\left({\mathrm{Samples}}_{{\rm{i}}}\right)\right)$$

Details about the 363 proteins, analytical range, data normalization and standardization are available online (www.olink.com).

### Identification of differentially abundant metabolites and proteins

To comprehensively characterize global alterations in metabolic and proteomic profiles across comparative cohorts, OPLS-DA was conducted via SIMCA software. Model validity was statistically verified through analysis of cross-validated residuals via CV-ANOVA (cross-validation analysis of variance), with a significance threshold defined at *p* < 0.05, confirming the discriminatory power between groups.

To identify differentially expressed features, metabolites and lipids were compared via the Mann‒Whitney U test, whereas Student’s t test was used for proteomics analysis. Additionally, the Benjamini‒Hochberg procedure was applied to control the false discovery rate for multiple comparisons. A *q*-value less than 0.05 was considered statistically significant. Furthermore, logistic regression analysis was performed to control for potential confounding factors, including the presence of diabetes, hyperlipidemia, anxiety and depression, and the use of anti-Parkinsonian drugs. The cutoff was set at an adjusted *p*-value < 0.1.

### Functional annotation of differential proteins

The list of DEPs was converted into the standard Entrez Gene Identifiers via the Gene ID Conversion Tool. GO, KEGG pathway, and Reactome pathway analyses were performed via DAVID. The GO enrichment bar plots, enrichment circle diagrams, and chord diagrams were generated via the analytical tools provided by the MetWare Cloud.

### Random forest analysis for feature selection

RF analysis was performed in the R statistical environment (accessed via RStudio version 2025.09.2) using the *random* *forest* package. To identify the most robust and stable predictors discriminating between groups, an iterative RF selection approach was implemented. The entire iterative process was made reproducible by setting a global random seed (set.seed(42)) prior to initiating the loop. A standard RF classification model was constructed over 999 independent iterations. For each iteration, the model was built using 500 decision trees (ntree = 500). For each feature, the mean and standard deviation of both its MDA and MDG scores were calculated after the 999 runs. The top 30 most important features, as ranked by the MDA and MDG scores, were visualized via bar charts created with the *ggplot2* package.

### Biomarker panel construction

The top 30 ranked molecules were subsequently subjected to binary logistic regression analysis in PASW Statistics (v18.0.0, IBM Corp.) via the forward stepwise method, yielding an optimized combinatorial biomarker panel. To obtain a robust and unbiased estimate of the model’s predictive performance, we employed an iterative leave-group-out cross-validation strategy. The complete dataset was randomly partitioned 1000 times into a training set (70% of the samples) and a hold-out test set (30% of the samples). In each of the 1000 iterations, a logistic regression model was built using the training data and subsequently used to predict the outcomes of the remaining test data. For each of the 1000 iterations, the AUC, sensitivity, and specificity were calculated on the hold-out test set. The mean and standard deviation of the AUC, sensitivity, and specificity across all 1000 cross-validation runs were calculated. Following this validation, a final logistic regression model was trained on the entire dataset (100% of the samples) to establish the definitive coefficients for the combined biomarker signature.

### Metagenomic sequencing

A total of 73 stool samples were obtained from participants (21 RBD-PD patients, 8 non-RBD-PD patients, 21 iRBD patients, and 23 healthy controls) enrolled in Cohort 2 following standardized clinical protocols. In brief, all participants were instructed to collect a fresh mid-portion stool sample using the provided sterile containers. The samples were then transported to the laboratory within 2 h on ice. Stool samples were aliquoted and stored at −80 °C until they were transported to Novogene (Beijing, China) for shotgun metagenomic sequencing. After initial sample inspection, DNA quality control was performed following Novogene’s standard QC workflow. Detailed information on whole-genome shotgun sequencing and data preprocessing is provided in the Supplementary Materials.

### Differential abundance analysis of gut microbial species and functional genes

The statistical significance of differential taxonomic and functional abundance in two-group comparisons was assessed in R. Data manipulation was performed via the ‘dplyr’ package. The normality of the distribution within each group was examined with the Shapiro–Wilk test. When both groups satisfied the normality assumption (Shapiro–Wilk *p* > 0.05), group means were compared via Welch’s two-sample t test; otherwise, the nonparametric Mann–Whitney U test was employed (including cases with insufficient sample sizes for the Shapiro–Wilk test). The resulting *p*-values were corrected for multiple comparisons via the Benjamini‒Hochberg procedure. The top 16 gut bacterial taxa whose relative abundances changed significantly in both RBD-PD and iRBD patients were identified by simultaneously satisfying *p* < 0.001 and |FC | > 1.5 in both the RBD-PD vs. Ctrl and iRBD vs. Ctrl comparisons. The top 38 most significantly dysregulated functional genes were identified by simultaneously meeting the criteria of *p* < 0.005 and |FC | > 1.5 in both the RBD-PD vs. Ctrl and iRBD vs. Ctrl comparisons.

## Supplementary information

Supplementary Materials

Supplementary Tables

Dataset 1

Dataset 2

## Data Availability

The proteomic data analyzed in this study are available at figshare (10.6084/m9.figshare.31007854). Proteomics data and protein mapping to UniProt identifiers and gene names were provided by Sinotech Genomics Co., Ltd. (Shanghai, China) and Olink. All datasets generated and/or analyzed during this study are provided in Supplementary Data 1. All R scripts used for logistic regression, random forest, and iterative leave-group-out cross-validation are available in Supplementary Data 2.
