# Supplementary material for: Distinct metabolomic and proteomic signatures in Parkinson’s disease patients with REM sleep behavior disorder
Source: Signal Transduct Target Ther. 2026 Mar 30;11:115. doi: 10.1038/s41392-026-02613-8 (PMC13035852; doi:10.1038/s41392-026-02613-8)
Supplement: Supplementary file 1 — Supplementary Materials [file 41392_2026_2613_MOESM1_ESM.docx]

Supplementary Materials for

Distinct metabolomic and proteomic signatures in Parkinson’s disease patients with REM sleep behavior disorder

Yaping Shao^1#^*, Jing Wang^2#^, Yaping Liu^2^, Yang Ni^1^, Zijiao Liu^1^, Yanli Li^3^, Qiqi Jia^1^, Qi Li^3^, Xiaolin Wang^3^, Tianbai Li^1^, Meichen Liu^1^, Shuining Zhang^1^, Yanming Guo^1^, Xisa Guo^1^, Dali Wang^4^, Yang Liu^5^, Cong Liu^6,7^, Huaibin Cai^8^, Yuping Ning^9,10^, Jihui Zhang^2,9^, Guowang Xu^3^*, Weidong Le^1,4^*

Correspondence to: wdle@sibs.ac.cn, xugw@dicp.ac.cn, alanna_s@foxmail.com

#: These authors contributed equally to this work.

**This PDF file includes:**

Materials and Methods

Figures. S1 to S7

**Other Supplementary Materials for this manuscript include the following:**

Supplementary Tables (Table S1 to S21)

Supplementary Data1 (All raw and normalized datasets generated and/or analyzed in this study.)

Supplementary Data2 (All R scripts used for logistic regression, random forest, and iterative Leave-Group-Out Cross-Validation during this study.)

Materials and Methods

Sample pretreatment for metabolomics analysis

Briefly, 100 μL of plasma was mixed with 400 μL of cold organic solvent (acetonitrile: methanol = 1:1, v/v; containing internal standards, **Supplementary Table 20**). After vortexing for 2 minutes, the mixture was centrifuged at 14,000 g (4°C) for 15 minutes. Two 200-μL aliquots of supernatant were then dried under vacuum at 4°C. For LC-MS analysis, the dried residues were reconstituted in 50 μL of solvent (25% acetonitrile in water), centrifuged at 15,000 g (4°C) for 15 minutes, and the clarified supernatants were transferred to glass vials. For GC-MS analysis, the dried extracts underwent a two-step derivatization process involving oximation followed by silylation prior to analysis.

Sample pretreatment for lipidomics analysis

Specifically, 40 μL of plasma was mixed with 300 μL of ice-cold methanol (containing internal standards, **Supplementary Table 21**) to precipitate macromolecular components. After vortexing for 1 minute, 1 mL of methyl tert-butyl ether was added, followed by vortexing for another 1 minute. Phase separation was induced by adding 250 μL of ultrapure water and vortexing for 1 minute. The mixture was incubated at room temperature for 10 minutes to achieve complete phase separation. Two 400-μL aliquots of the upper organic phase were transferred to 1.5 mL centrifuge tubes and dried under vacuum at 4°C. The dried lipid extracts were reconstituted in 25 μL of solvent (dichloromethane: methanol = 2:1, v/v), then diluted with 50 μL of 5 mM ammonium acetate solution (acetonitrile: isopropanol: water = 65:30:5, v/v/v). After vortexing and centrifugation, the supernatant was subjected to lipidomics analysis.

Metabolomic profiling

LC-MS analyses were performed using an Ultra Performance Liquid Chromatography (UPLC, Waters, Manchester, UK) coupled to a TripleTOF™ 5600+ mass spectrometer (SCIEX, Marlborough, MA). For positive ionization mode (ESI+) mode, separation was achieved on an ACQUITY UPLC BEH C8 column (2.1 × 50 mm, 1.7 μm). Negative ionization mode (ESI-) analysis employed an ACQUITY UPLC HSS T3 column (2.1 × 50 mm, 1.8 μm). Detailed chromatographic gradients and mass spectrometric acquisition parameters are described in our previous study (*Mol Neurodegener.* 16, 4 (2021)).

GC-MS analysis was conducted on a QP 2010Plus GC-MS system equipped with an AOC-20i autosampler (Shimadzu, Kyoto, Japan). The complete temperature programming protocol and mass spectrometric detection settings have been documented previously (*Signal Transduct Target Ther*. **8**, 334 (2023)).

Lipidomic profiling

Lipidomics analysis was performed on a hyphenated LC-MS system equipped with a Nexera LC-40 HPLC (Shimadzu, Kyoto, Japan) and a ZenoTOF 7600 MS (SCIEX, Marlborough, MA). Lipid separations were performed on an ACQUITY UPLC BEH C8 column (2.1 × 100 mm, 1.7 μm) using both ESI+ and ESI- ionization modes, enabling high-coverage profiling of structurally diverse lipid species. Detailed chromatographic and mass spectrometric parameters refer to our previous study (*Signal Transduct Target Ther*. **8**, 334 (2023)).

Data quality control

To monitor the stability and reproducibility of the entire analytical workflow, a pooled quality control (QC) sample was prepared by combining equal aliquots (10 µL) from each plasma sample. The resulting pool was thoroughly mixed, aliquoted, and processed using the same sample preparation protocol as the study samples. To mitigate the impact of systematic signal drift on inter-group comparisons, the injection order of samples from the three groups was randomized. A QC sample was injected after every 18 study samples throughout the analytical batch (**Supplementary Fig.2a**). Data quality assessment was conducted as follows: PCA was performed in SIMCA (V14.1.0.2047, Umetrics AB) to detect potential systematic analytical drift in QC samples throughout the analytical sequence. Concurrently, the RSD of each quantified metabolite in the QC replicates was calculated; an RSD values < 30% considered to indicate acceptable analytical precision.

Internal standard calibration

An optimal internal standard (IS) was selected for each individual feature (metabolite or lipid) using the QC samples. Specifically, the raw peak area of each feature was calibrated against each candidate IS, and the RSD of the feature’s calibrated area across all QC samples was calculated. The IS that yielded the lowest RSD for a given feature was selected as its optimal standard for calibration. Finally, this feature-specific IS correction was applied to all analytical samples, generating semi-quantitative concentration values (in µg/mL) that were used for statistical analysis.

Metabolic pathway enrichment analysis

Differential metabolites were systematically annotated against the Human Metabolome Database (HMDB) to establish standardized HMDB identifiers, followed by comprehensive pathway enrichment analysis using the Pathway Analysis module of MetaboAnalyst 5.0. This analytical workflow employed: i) over-representation analysis via hypergeometric test, ii) topological assessment using relative-betweenness centrality metrics, and iii) pathway mapping against the Kyoto Encyclopedia of Genes and Genomes (KEGG) database (released in Dec. 2024).

Metagenomic sequencing

For library preparation, 1 μg of high-quality genomic DNA was fragmented to approximately 350 bp using a Covaris ultrasonicator. Fragmented DNA underwent end repair, A-tailing, adaptor ligation, size selection, and PCR amplification. Library quality and insert size distribution were examined using AATI analysis, and quantification was performed via qPCR (effective concentration >3 nM). Libraries passing QC were pooled according to target sequencing depth and subjected to Illumina NovaSeq paired-end 150 bp whole-genome shotgun sequencing.

Raw reads were filtered using fastp to remove adaptor contamination, low-quality sequences, and reads containing >10% ambiguous bases. High-quality reads were aligned to the human reference genome using Bowtie2 to remove host contamination. Reads mapping to the host genome were discarded prior to downstream metagenomic analysis.

Metagenomic profiling and data preprocessing

High-quality non-host reads were assembled using MEGAHIT, and open reading frames (ORFs) were predicted using MetaGeneMark. Non-redundant unigenes were constructed using CD-HIT (95% identity), and clean reads were mapped back to unigenes using Bowtie2 to obtain gene-level read counts. Taxonomic annotation was performed by aligning unigene protein sequences to the MicroNR database using DIAMOND blastp (e-value ≤ 1×10⁻⁵), followed by lowest common ancestor (LCA) assignment via MEGAN. Species-level, genus-level, and higher-rank taxonomic abundance tables were generated by summing the abundances of genes assigned to each taxon.

The functional potential was assessed by aligning unigenes to the KEGG database using DIAMOND blastp (e-value ≤1×10⁻⁵). KO assignments with the best-hit score were retained, and gene abundances were summed to generate KO-level functional profiles. KO terms were further mapped to KEGG pathways and modules when required for interpretation. Taxonomic relative abundance tables and KO abundance matrices were normalized across samples using total-sum scaling. To integrate microbial metagenomic features with targeted plasma metabolomics, all microbial taxa and KO terms were filtered to remove features with extremely low prevalence.


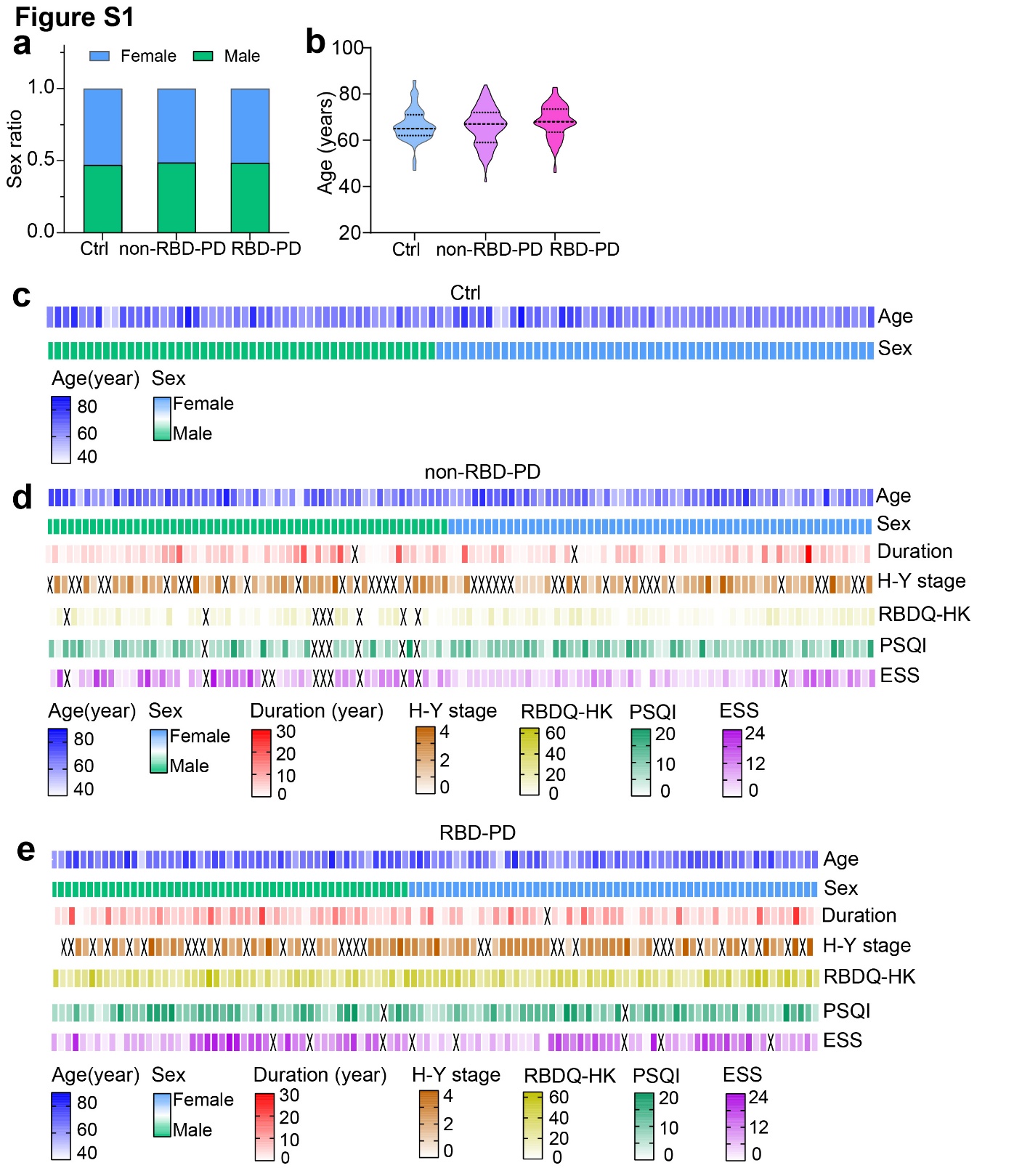


Figure. S1.

**Participant recruitment and clinical characteristics of subjects.** **a-b** Sex ratio and age distribution of subjects. The dashed lines represent the median and quartiles. **c-e** Clinical characteristics of subjects. X indicates missing values for this parameter. H-Y, Hoehn-Yahr; PSQI, Pittsburgh sleep quality index; RBDQ-HK, REM sleep behavior disorder questionnaire - Hong Kong; ESS, Epworth sleepiness scale.


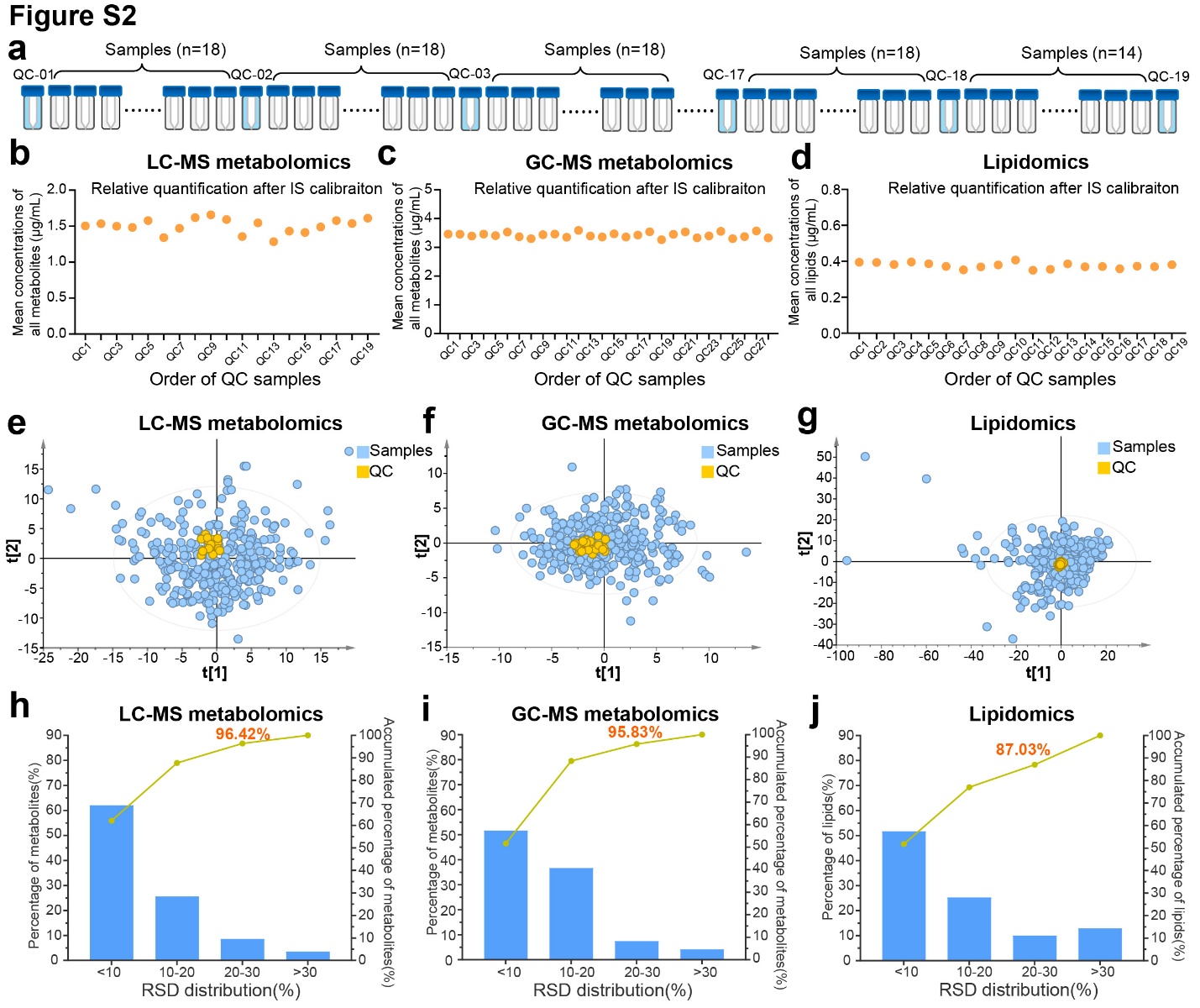


Figure. S2.

**Quality control in three analytical platforms.** **a** Running sequence of the samples in metabolomics and lipidomics analysis. **b-d** Mean concentrations of all metabolites detected in quality control (QC) samples in LC-MS-based metabolomics, GC-MS-based metabolomics and LC-MS-based lipidomics analyses. **e-g** Principal component analysis of all plasma and QC samples. QC samples cluster tightly on the center of the score plot, indicating high analytical robustness throughout the instrumental analysis. **h-j** Relative standard deviation (RSD) distribution of all detected metabolites/lipids in QC samples for evaluating method repeatability.


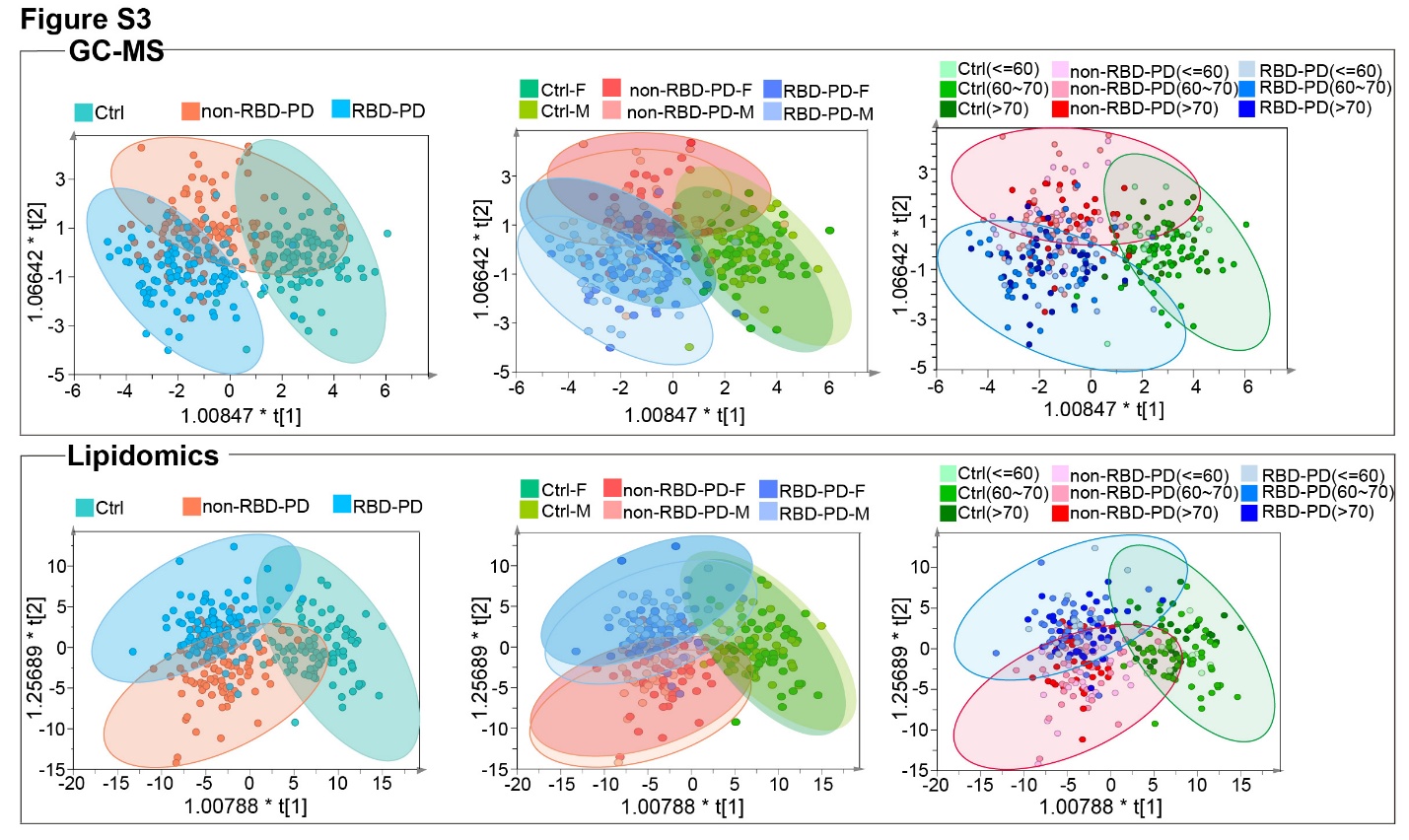


Figure. S3.

**OPLS-DA model of two PD subgroups and control individuals based on GC-MS metabolomics and LC-MS lipidomics.** Model parameters for GC-MS-based metabolomics, R2Y = 0.486, F factor = 6.58, *p* value = 1.80E-023. Model parameters for LC-MS-based lipidomics, R2Y = 0.551, F factor = 5.87, *p* value = 3.23E-020.


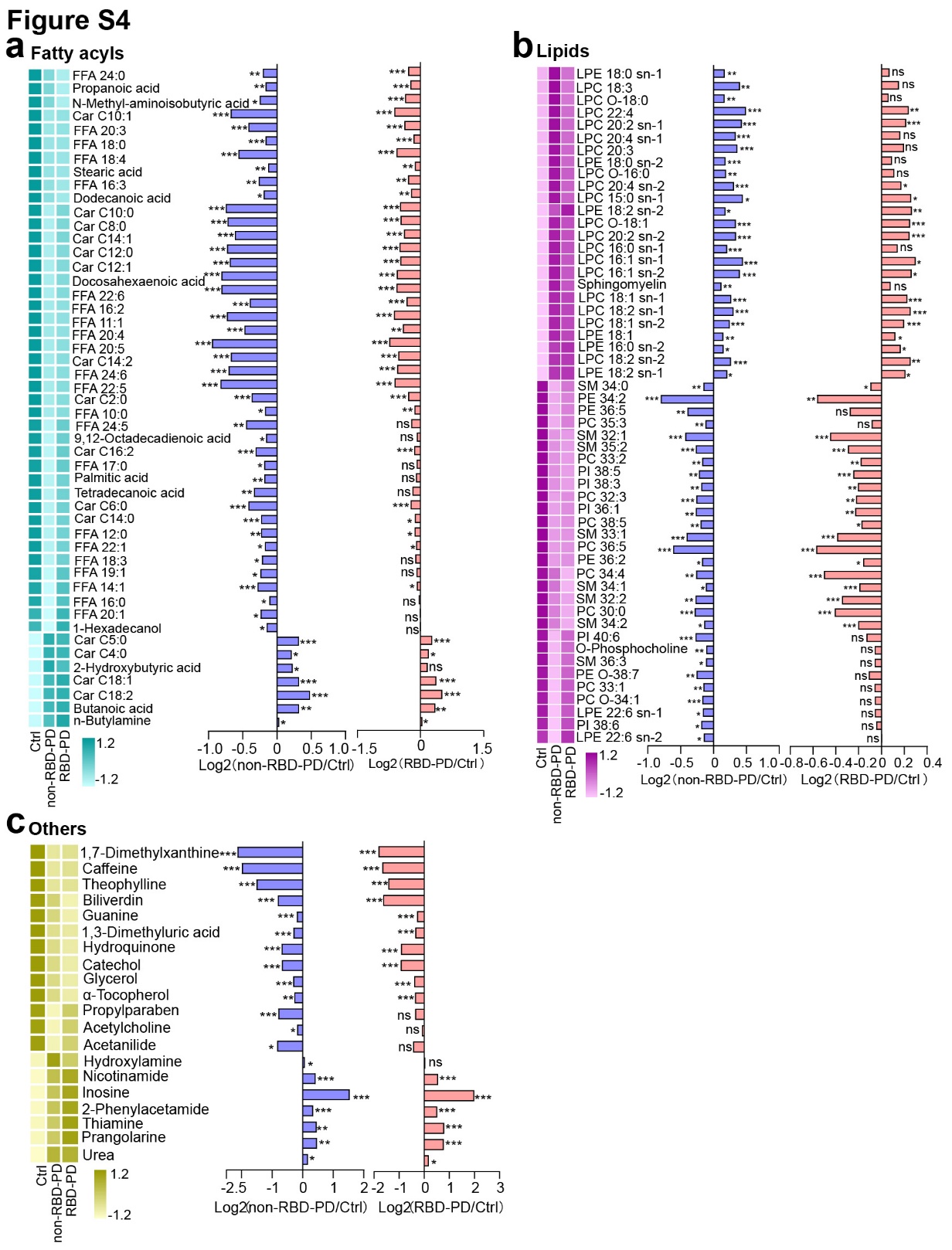


Figure. S4.

**Heatmap of relative concentrations of PD differential metabolites and fold-changes comparing two PD subgroups versus healthy controls. a** fatty acyl, **b** lipids, **c** other chemicals. FFA: fatty acid; Car: acylcarnitine; LPE: lysophosphatidylethanolamine; LPC: lysophosphatidylcholine; PE: phosphatidylethanolamine; PC: phosphatidylcholine; PI: phosphatidylinositol; SM: sphingomyelin.


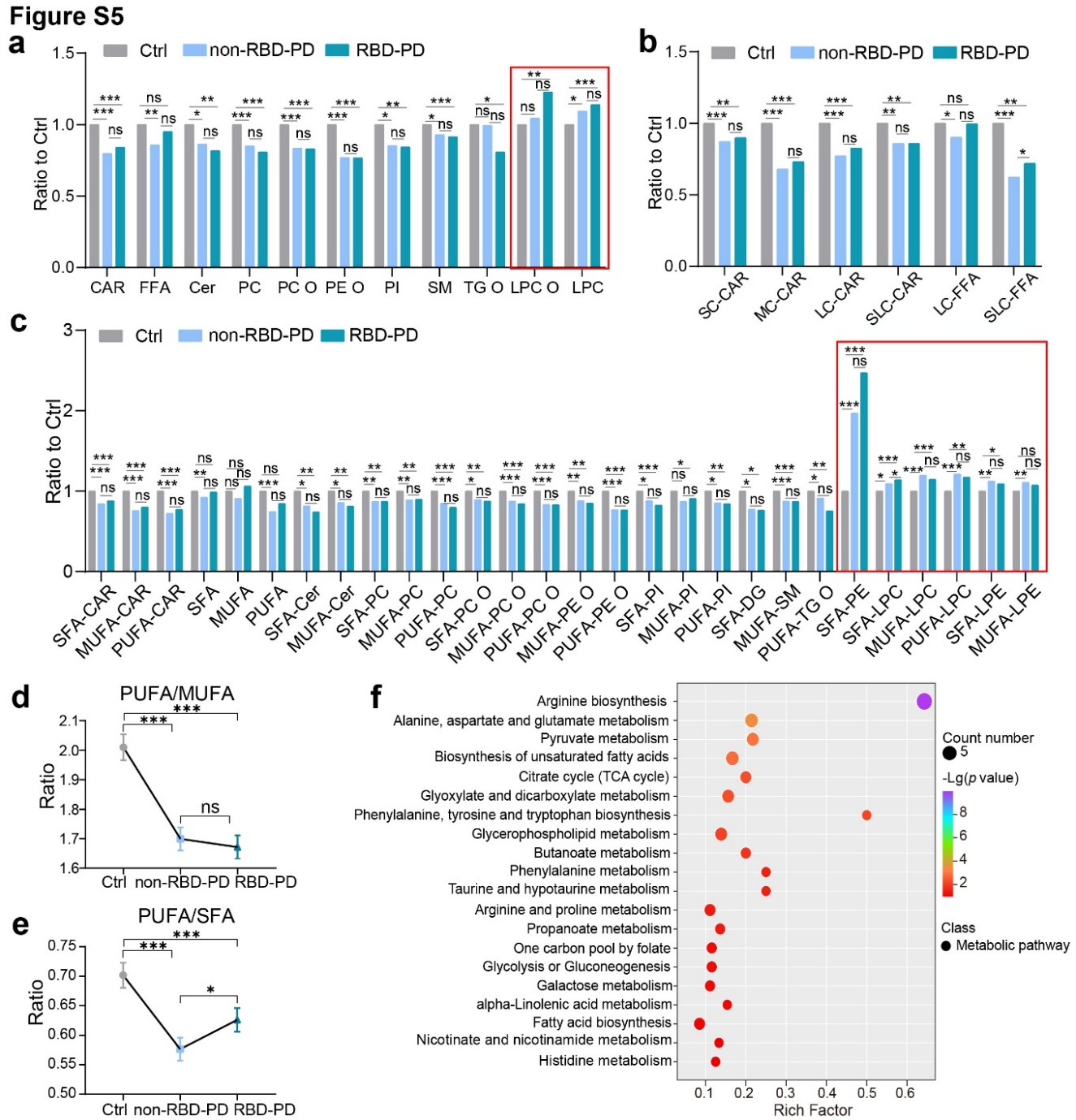


Figure. S5.

**Alterations in lipid and metabolic pathways in PD. a** Changes in total level of each lipid class. CAR, acylcarnitine; FFA, fatty acid; Cer, ceramide; PC, phosphatidylcholine; PC O, alkyl-acyl PC; PE O, alkyl-acyl phosphatidylethanolamine; PI, phosphatidylinositol; SM, sphingomyelin; TG O, alkyl-acyl triacylglycerol; LPC, lysophosphatidylcholine; LPC O, alkyl-acyl LPC. **b** Alterations in total levels of acylcarnitines and fatty acids with varying chain lengths. SC, short-chain; MC, medium-chain; LC, long-chain; SLC, super long-chain. **c** Alterations in total levels of acylcarnitines and fatty acids with varying degrees of saturation. SFA, saturated fatty acids; MUFA, monounsaturated fatty acid; PUFA, polyunsaturated fatty aicd. **d-e** Alterations in ratio of PUFA to MUFA and SFA. Ctrl, n = 102; non-RBD-PD, n = 110; RBD-PD, n = 102. **f** Enrichment analysis of disordered metabolic pathways in PD. *: 0.01 < *q* < 0.05, **: 0.001 < *q* < 0.01, ***: *q* < 0.001. The *q* value represents the adjusted *p* value.


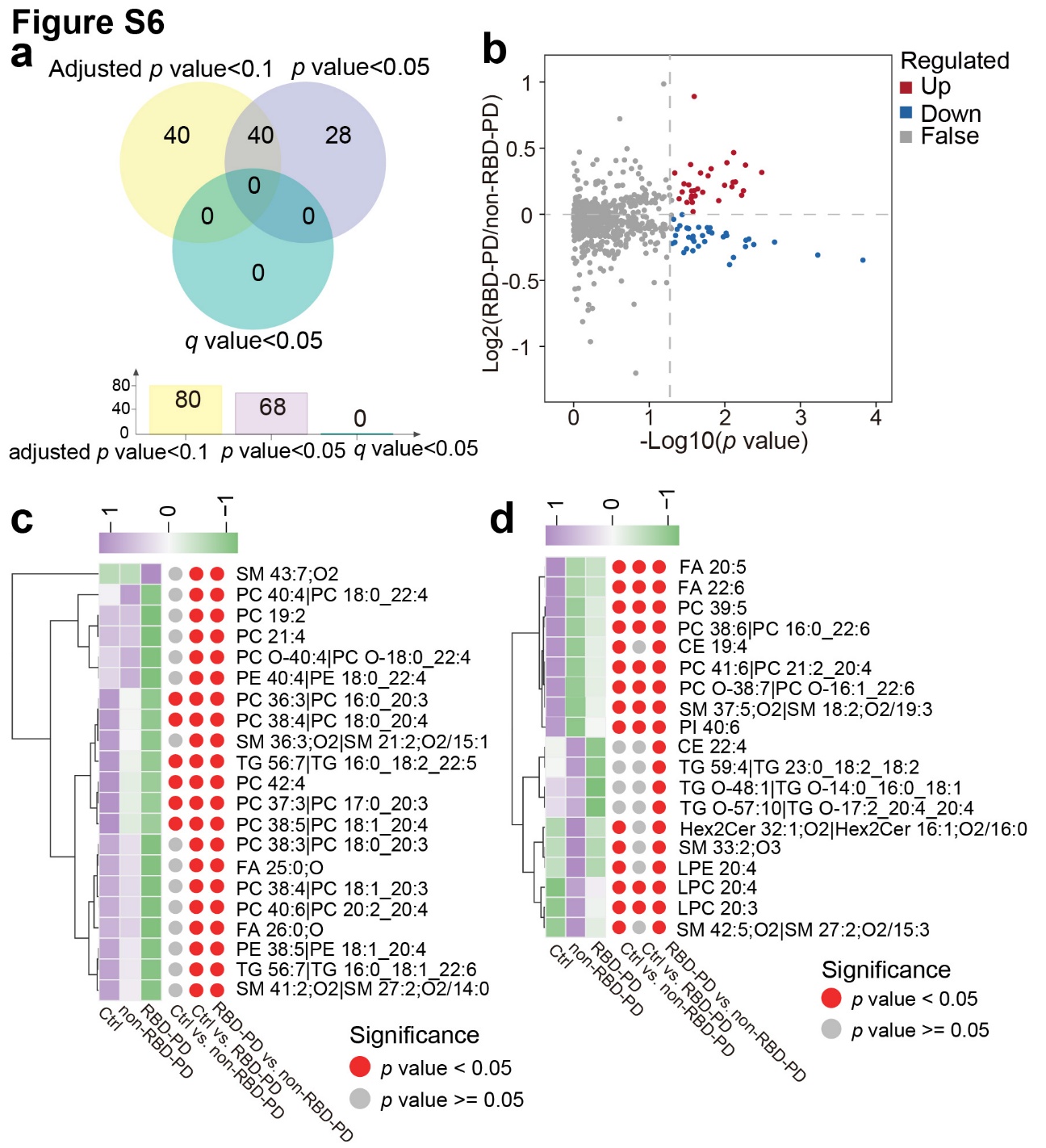


Figure. S6.

**Lipidomic differences between PD patients with and without RBD. a** Venn diagram of differential lipids associated with RBD-PD in intergroup comparisons. **b** The volcano plot visualizes significantly changed differential lipids in RBD-PD compared to non-RBD-PD (based on *p* value). **c** Heatmap of DELs exhibiting linear level changes across Ctrl, non-RBD-PD patients, and RBD-PD patients. **d** Heatmap of lipids exhibiting V-shaped or inverted V-shaped concentration changes across Ctrl, non-RBD-PD patients, and RBD-PD patients. The color scale in the heat map represents the Z-score. The *p* values presented were unadjusted for multiple comparisons.


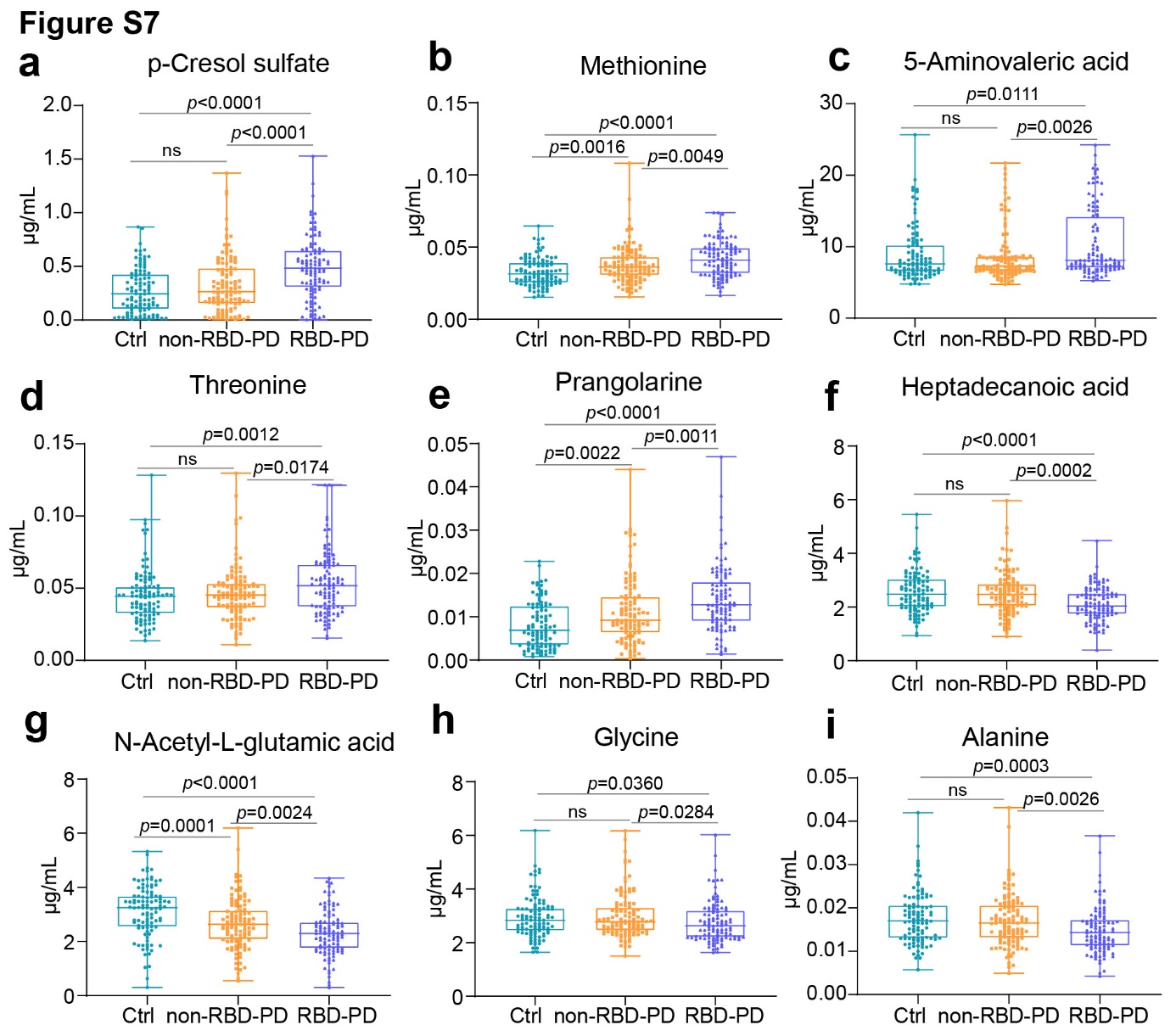


Figure. S7.

**Box plots of relative concentrations for metabolites in the biomarker model across three groups.** *: 0.01 < *p* < 0.05, **: 0.001 < *p* < 0.01, ***: *p* < 0.001.

Tables S1-S21. (separate file)

**Table S1.** Detailed characterization information for metabolite identification via LC-MS.

**Table S2.** Detailed characterization information for metabolite identification via GC-MS.

**Table S3.** Detailed characterization information for lipid identification via LC-MS.

**Table S4**. Statistics of differential metabolites in non-RBD-PD group compared to Ctrl.

**Table S5**. Statistics of differential lipids in non-RBD-PD group compared to Ctrl.

**Table S6**. Statistics of differential proteins in non-RBD-PD group compared to Ctrl.

**Table S7**. GO annotations for DEPs of non-RBD-PD.

**Table S8.** KEGG pathway enrichment analysis for DEPs of non-RBD-PD.

**Table S9.** Statistics of differential metabolites in RBD-PD group compared to non-RBD-PD.

**Table S10.** Statistics of differential proteins in RBD-PD group compared to non-RBD-PD.

**Table S11.** GO annotations for DEPs of RBD-PD.

**Table S12**. Reactome pathway enrichment analysis for DEPs of RBD-PD.

**Table S13.** Parameters and predictive performance of the binary logistic regression model to distinguish RBD-PD from non-RBD-PD in cohort 1.

**Table S14**. Parameters and predictive performance of the binary logistic regression model to distinguish RBD-PD from controls in cohort 1.

**Table S15.** Statistics of significantly differential metabolites in RBD-PD in two independent cohorts.

**Table S16.** Parameters and predictive performance of the binary logistic regression model to distinguish RBD-PD from non-RBD-PD in cohort 2.

**Table S17.** Parameters and predictive performance of the binary logistic regression model to distinguish RBD-PD from controls in cohort 2.

**Table S18.** Statistics of differentia gut microbial species in RBD-PD and iRBD groups compared to Ctrl.

**Table S19.** Statistics of differentia gut microbial functional genes in RBD-PD and iRBD groups compared to Ctrl.

**Table S20.** Concentrations of internal standards in metabolomics analysis.

**Table S21**. Concentrations of internal standards in lipidomics analysis.

Data S1. (separate file)

Raw data of LC-MS ESI+;

Calibrated data of LC-MS ESI+;

Raw data of LC-MS ESI-;

Calibrated data of LC-MS ESI-;

Raw data of GC-MS;

Calibrated data of GC-MS;

NPX data of proteomics;

Calibrated data in cohort 2;

KO_ID data in cohort 2;

OUT data in cohort 2.

Data S2. (separate file)

R Script for confounding factor adjustment;

R Script for random forest;

R Script for cross-validation.
